# Recent Progress in Designing Nanomaterial Biohybrids for Artificial Photosynthesis

**DOI:** 10.3390/nano15100730

**Published:** 2025-05-12

**Authors:** Sampathkumar Jeevanandham, Subramaniyan Ramasundaram, Natarajan Vijay, Tae Hwan Oh, Subramanian Tamil Selvan

**Affiliations:** 1 Molecular Science and Engineering Laboratory, Amity Institute of Click Chemistry Research and Studies, Amity University, Noida 201313, India; sampathkumarj4@gmail.com; 2 School of Chemical Engineering, Yeungnam University, Gyeongsan 38541, Republic of Korea; ramasundaram79@hotmail.com (S.R.); jmcvijay@gmail.com (N.V.); 3 Department of Interdisciplinary Sciences, National Institute of Food Technology and Entrepreneurship Management, Sonipat 131028, India; 4 Azion Global Pte. Ltd., 622 Bukit Batok Central, #11-504, Singapore 650622, Singapore

**Keywords:** artificial photosynthesis, nanomaterial biohybrids, photocatalysts, solar energy conversion, CO_2_ fixation

## Abstract

In natural photosynthesis, solar energy is utilized to convert water and CO_2_ into energy-rich compounds. However, in practice, the maximum quantum efficiency of natural photosynthesis is limited to 6.0%. Conversely, artificial photosynthesis (AP) systems utilize solar energy to convert CO_2_ into biosynthetic solar fuels and value-added chemicals. To mimic natural photosystems, AP integrates light-harvesting chemical catalysts with the enzyme-mediated biological catalysis occurring in microorganisms. Similar to solar energy-based optoelectronic power sources, AP has also been recognized as a promising option for reducing carbon emissions generated by the fossil fuel-based power sector. Typical quantum efficiency of AP is 5–10%; in some cases, it exceeds 20%. Recent advancements have focused on nanomaterial biohybrids (NBHs), combining nanomaterial-based photocatalysts/photosensitizers with microorganisms/enzymes for enhanced oxidation/reduction reactions. The synergistic interaction between nanomaterials and microorganisms, facilitated by their comparable size and tunable surface properties, enables improved solar energy absorption, charge separation, and conversion. NBHs offer a versatile platform for sustainable solar energy harvesting and conversion, overcoming the limitations of natural and fully abiotic photosynthesis systems. This review highlights recent breakthroughs in diverse platforms of sunlight and visible light-driven NBH-based AP systems for CO_2_ fixation, H_2_ production, water splitting, and value-added chemical synthesis. The synthesis strategies, operating mechanisms, and challenges are highlighted.

## 1. Introduction

Sunlight-driven natural photosynthesis converts carbon dioxide (CO_2_) and water into carbohydrates and oxygen, and plays a crucial role in maintaining the carbon cycle. In natural photosynthesis, the light photosystem harvests sunlight and performs photochemical conversion reactions via redox reactions, electron migration, charge separation, etc. Then, the energy carriers are utilized in the dark photosystem, where atmospheric CO_2_ is converted into carbohydrates and nutrients. However, in natural photosynthesis, limitations in light absorption constrain the conversion efficiency of solar energy to biomass. Solar conversion efficiency is estimated based on quantum efficiency, which refers to the proportion of absorbed photons converted into stable photoproducts. Under optimum conditions, the photosynthetic organisms operate at a quantum efficiency close to 100%. However, the pigments in plants such as chlorophyll and carotenoids can only intercept the visible region (400 to 700 nm), which amounts to 42–43% of the solar spectrum. Based on chlorophyll band edge absorption, the theoretical limit of solar energy conversion is ~12%. Losses associated with light conversion, such as overpotentials and respiration, reduce efficiency below the theoretical maximum. The solar conversion efficiency in plants forming three- and four-carbon compounds in the first step of photosynthesis amounts to 4.6 and 6.0%, respectively. In temperate and tropical crops, the solar conversion efficiency is limited to less than 1% [1].

To address the limitations of natural photosynthesis in harnessing solar energy, artificial photosynthesis (AP) systems were developed. AP systems harness sunlight to convert CO_2_ into fundamental feedstock (carbon monoxide, methane, formate, and acetate), valuable chemicals, and transportation fuels. These photochemical conversion reactions mostly occur under mild reaction conditions (ambient temperature and pressure) and do not require additional activation energy. In practice, the quantum efficiency of AP typically ranges from 5 to 10%. However, in some cases, the quantum efficiency of AP can exceed 20% [2].

CO_2_ is the primary greenhouse gas contributing to global warming and climate change. Global warming has led to climate change, causing sudden rises in sea level, unseasonal heavy rains, and huge natural disasters. The issue of carbon emissions from fossil fuel-based energy generation is a driving force for the growth of AP-based carbon-neutral fuel generation technology. Along with solar energy-based optoelectronic power generation methods, AP contributes to the reduction of carbon emissions and sources, maintaining the carbon cycle [3,4]. It is estimated that around 80–85% of global energy needs rely on fossil fuels, whose emissions have raised the level of atmospheric CO_2_ to 427 ppm [5]. AP-derived carbon-neutral fuels are estimated to be capable of fixing 40% of the energy scarcity prevailing in the global transportation industry [6].

In 1972, Fujishima et al. reported the photocatalytic properties of titanium dioxide (TiO_2_), a seminal discovery in the field of semiconductor photocatalysis [7]. Subsequent exploration of photocatalytic activity in other transition metal oxides-based semiconductor photocatalysts has propelled the development of AP as an effective technology for sunlight-driven photochemical conversion of CO_2_ into chemicals and fuels [8,9]. These pioneering studies have further promoted extensive research to enhance the performance of AP systems.

AP systems synergistically integrate the physicochemical properties of synthetic photocatalytic/photosensitizing nanomaterials (PNMs) with enzyme-mediated biological catalysts [10]. Here, nanomaterials refer to nanoparticles (NPs), nanoclusters (NCs), or quantum dots (QDs). Among various AP systems, the combination of PNMs and microbes is considered more advantageous than PNMs–enzyme hybrids. The expensive processes required for the isolation and purification of enzymes drastically limit their integrated application with PNMs. Conversely, microbial cells can easily multiply through self-replication and can adjust to their growth environment through self-repair and adaptability processes. Additionally, the combinatorial role of metabolic functions and complex enzyme systems in chemical conversion reactions exceeds the functional capacity of PNMs–enzyme hybrids [11,12]. PNMs–microorganism combinations bring the best attributes of whole-cell biocatalysts and PNMs. Specifically, self-healing, replicating, and specificity of microbes are integrated with the solar light-harvesting capabilities of PNMs. Amplification of microbial cells proportionally increases the number of active sites in these integrated catalytic platforms. Enzymes in the native cellular environment lower the activation energy barrier and offer selectivity. When exposed to an appropriate light source, PNMs become excited and generate electrons and holes at their outer surface. These charge carriers then migrate into microbial cells, where they participate in redox reactions with various molecules, ions, and radicals under ambient conditions. Such redox reactions yield fine chemicals, fuels, and intermediates. Typically, CO_2_ and H^+^ are converted into acetate and H_2_, respectively. The holes oxidize electron donors, such as H_2_O, and sacrificial reagents into oxidized products.

In the field of materials chemistry, AP systems serve as platforms for nanomaterial biohybrids (NBHs) [13,14,15]. These NBHs outperform natural photocatalytic platforms in diverse key aspects, including their structural simplicity, charge transportation controllability within photosystems, and a broader range of spectral absorption. In contrast, biological systems function through complex, intricate, and energy-intensive mechanisms. Inorganic nanomaterials—including metals, metal oxides, metal organic frameworks [MOFs], and metal chalcogenides—along with organic and polymeric counterparts capable of absorbing components of broadband solar irradiation, have been used as photothermal nanomaterials (PNMs). PNMs offer tunable morphology, band gaps, broadband light harvesting, surface charge, chemical composition, and substantial contact area. The size scale of PNMs mostly matches the size of microbial cells.

Some of the commonly used inorganic/semiconductor-based PNMs include TiO_2_ and cobalt phosphate (CoP) NPs, cadmium sulfide (CdS), cadmium selenide (CdSe), and indium phosphide (InP) QDs, and gold (Au) NCs. Among various PNMs, CdS (2.4 eV, 517 nm) was utilized to a larger extent. The photoexcited electrons generated by CdS thermodynamically favor integrated microbial redox reactions, including the regeneration of nicotinamide adenine dinucleotide (NADH) and nicotinamide adenine dinucleotide phosphate (NADPH), as well as the reduction of CO_2_ to formate. Because of their high biocompatibility, organic conjugated polymers such as oligofluorene (OF) and polythiophene (PTP) were also used in diverse NBH platforms. Photosynthetic and non-photosynthetic microorganisms, including bacteria, yeast, and enzymes, were integrated as biological counterparts. *Escherichia coli (E. coli)*, *Moorella thermoacetica (M. thermoacetica)*, *Sporomusa ovata (S. ovata)*, *Methanosarcina barkeri (M. barkeri)*, *Cupriavidus necator (C. necator)*, and *Rhodopseudomonas palustris (R. palustris)* are the typical bacterial species used as whole-cell microbial catalysts for preparing NBHs [16,17,18,19].

Natural sunlight, a free and clean energy source, is highly preferred as a direct energy input for various practical applications. In addition, lamps simulating the full spectrum or specific components, such as visible light, near-infrared (NIR), and ultraviolet A (UVA) radiation, are also used. Microbial cells are known to function effectively under solar irradiation at an intensity of 100 mW/cm^2^ [20]. However, sunlight’s intensity varies with weather conditions, seasons, and the duration of daylight. It is important to note that the UV component of sunlight can be detrimental to microbial cells. To address these limitations, visible light-emitting diodes (LEDs) equipped with cut-off filters eliminate short-wavelength light, and UV-cut xenon lamps are commonly employed as standard light sources. These artificial light sources selectively transmit specific wavelengths of light, enabling more controlled and biocompatible light-harvesting conditions [4]. By integrating NBHs with these light sources, several AP systems have been developed for the production of chemicals and fuels through CO_2_ conversion and water splitting.

Recent efforts have focused on improving the efficiency and sustainability of these AP systems. Genetic engineering techniques have been employed to increase the photo-tolerance of microbial cells, enabling NBHs to operate effectively under high-intensity light sources [21,22]. To mitigate energy loss caused by multiple intermediate electron transport steps, tuning the band gap of PNMs has been explored as a viable solution. Reactive oxygen species (ROS), known for their cytotoxic effects, present an additional challenge. Therefore, PNMs with low ROS generation and higher ROS scavenging potential are preferred for constructing robust NBHs. Efforts continue to address challenges related to product selectivity, thereby expanding the range of chemical products and improving overall yield in AP processes.

This review highlights recent advances in the development of NBHs for AP, focusing on strategies to enhance solar and visible light harvesting efficiency, stability, scalability, and chemical productivity. Given the interdisciplinary scope of this topic, an overview of the cellular distribution of PNMs and associated photoelectron transfer pathways is provided before the detailed discussion.

## 2. Internalization and Compartmentalization of PNMs in Microbial Cells and Mechanisms of Photoelectron Transfer

**Scheme 1:**
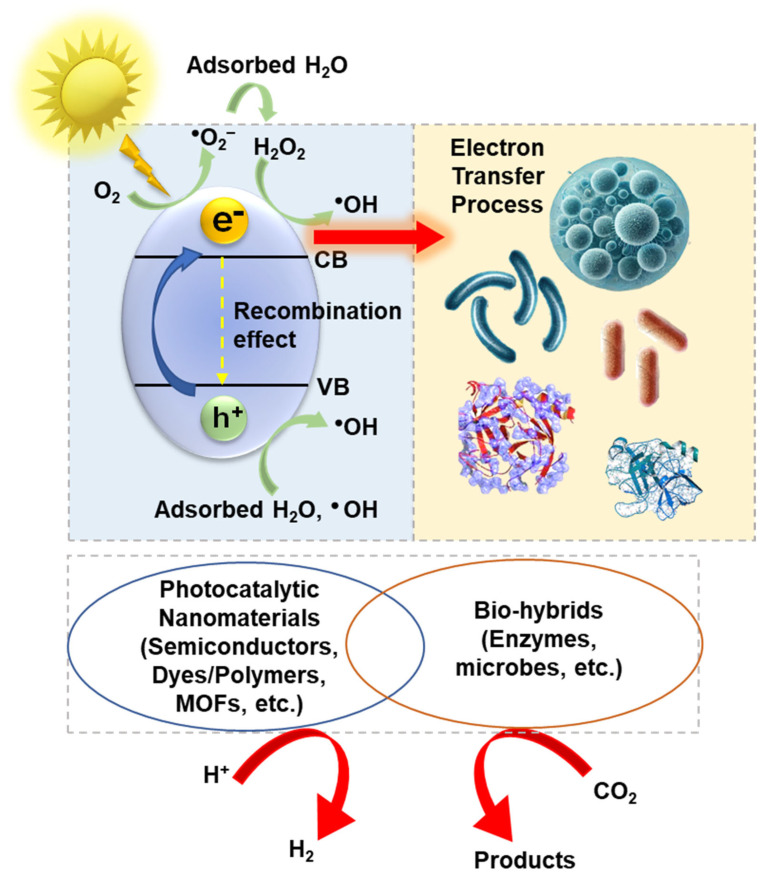
Schematic illustration depicting the synergistic role of PNMs–microorganism biohybrid systems.

NBHs based on PNMs represent a promising approach for addressing energy and environmental challenges through the synergistic fusion of nanotechnology and microbiology (Scheme 1). However, there are certain crucial factors affecting the AP performance of NBHs, such as surface charge, shape/size, and precise location of the PNMs in the cellular environment (intracellular or extracellular region), and tunability of the band gap of PNMs [3]. These factors can decide the selectivity and compatibility of PNMs in conjunction with several microorganism counterparts. Spatial distribution of PNMs within the cellular environment significantly influences the cross-membrane electron transport. The effectiveness of electron transport from PNMs to the intracellular domain determines the quantum of energy utilized by microbial cells. When the PNMs are simply suspended with living microbial cells, they are predominantly localized on the extracellular surface, such as the cell wall and outer cell membrane. These types of NBHs are known as extracellular hybrids, where photogenerated electrons are transported through various routes, involving cellular electroactive components and electron mediators or energy carriers. Extracellular hybrids require the inclusion of electron mediators for optimal performance. In the advanced hybrid systems, PNMs are distributed within the intracellular space, potentially enhancing direct electron transfer mechanisms and increasing the overall efficiency of energy conversion [4]. These NBHs are called intracellular hybrids.

The size of PNMs also plays a crucial role in determining their compartmentalization and spatial distribution within microbial cells. PNMs can be anchored in various cellular compartments, including cross-membranes, the periplasmic space, and the cytoplasm; (i) PNMs with diameters ≥ 20 nm typically localize at the cross-membrane interface, (ii) PNMs around 10 nm in size can move into the periplasmic space, and (iii) smaller PNMs, with diameters of 3–5 nm or less, are capable of entering the cytoplasm, where they dislocate and interact within the intracellular environment. Among the various properties of PNMs, including size, shape, and surface functional groups, size is a critical factor influencing cellular penetration. PNMs with a size of 25–50 nm exhibit high levels of cellular internalization. However, when the particle size exceeds 50 nm, the extent of internalization is significantly reduced, and the particles tend to adhere to the cell wall. Due to their tendency to aggregate, PNMs in suspension often accumulate on the cell surface rather than being internalized. In particular, amorphous materials such as polymeric and organic semiconductors tend to adhere to the cell wall or outer membrane. When PNMs are localized on the cell wall, charge transfer occurs through the wall. In contrast, if the PNMs are internalized, the cell wall must remain sufficiently stable to allow effective light transfer. Therefore, microbial cell walls are often engineered to enhance their structural stability and facilitate this process.

Internalization of PNMS occurs via various mechanisms. Internalization of ultra-small (below 10 nm) PNMs can occur via passive translocation, which does not exert a toxic effect, and the cell viability remains intact. In a certain medium, PNMs can loosen the membrane and allow PNMs to translocate into the cell. PNMs can also be internalized into the cell via endocytic mechanisms, where the PNMs enter cells via membrane wrapping. The PNMs are drawn into the vesicles and engulfed inside the cell. Based on their size and surface groups, PNMs can be internalized via various endocytic mechanisms, including phagocytosis, pinocytosis, macropinocytosis, and receptor (or clathrin/caveolae)-mediated endocytosis. Clathrin-mediated endocytosis typically forms vesicles ~120 nm in diameter, while caveolae-mediated endocytosis forms smaller vesicles of around 60 nm. Thus, receptor-mediated endocytosis pathways play a vital role in the internalization of PNMs. In the other endocytosis mechanisms, the size of vesicles (>1 µM) differs based on the size of particles to be engulfed. Biomineralization is another pathway of mineralization where the PNMs are synthesized inside the cell. The typical example is the formation of CdS nanoparticles from Cd^2+^ fed externally, and cysteine (Cys) produced inside the microbial cell.

The intracellular distribution of PNMs promotes the simultaneous generation of photoelectrons and efficient cross-membrane electron transport. In contrast, PNMs located on the extracellular surface are subject to greater energy losses due to multi-step electron transfer processes. Intracellular localization minimizes these energy losses by enabling direct electron transfer from PNMs to enzymes involved in cellular metabolism. However, this configuration may also introduce energy loss through side reactions within the cell and affect the overall AP efficiency NBHs [23]. The surface charge of PNMs significantly influences their adhesion to microbial cells. Positively charged PNMs exhibit a stronger affinity for the negatively charged cell membrane, stabilizing the PNM–cell interface through electrostatic interactions. This enhanced adhesion facilitates more efficient energy transfer across the cell membrane.

The efficiency of the photoelectron transfer within PNMs and NBHs plays a crucial role in determining the rate of CO_2_ bio-reduction, enzymatic H_2_ generation, and other enzymatic redox-active cofactors. The photoelectrons can be delivered in two mechanisms, either the direct or indirect mode, to the electron transfer process. The direct method of electron transfer involves transferring photogenerated electrons directly onto the biohybrids’ active central component, which eventually produces value-added products and aids in several CO_2_-fixing pathways. In comparison, the indirect method of electron transfer mechanisms involves more complex pathways of transferring the photoexcited electrons to the microbes/enzymes via designated redox mediator molecules or intermediates [24,25].

The potential cytotoxicity of PNMs toward microbial cells is a critical factor that must be considered when constructing NBHs. When PNMS comes in contact with microorganisms, interaction occurs due to collision. Then, adhesion occurs via electrostatic interactions and binding molecules on the surface. As mentioned above, adhered molecules can be internalized and compartmentalized inside the microbial cell. Surface adherence and internalization reduce the distance between the PNMs and cellular components. Sustained contact with PNMs, acting as exogenous agents, can induce cytotoxic effects in microbial cells, impairing their viability and proliferation. Consequently, this reduces the overall efficiency of AP systems. Biocompatible PNMs without strong antimicrobial activity are preferred for constructing efficient AP hybrids. The toxic effect of PNMs on microorganisms is addressed by designing a compatible size, surface area, surface properties, and compartmentalization in a specific location of the microbial cell. The ratio of the surface area of PNMs to microorganisms plays a vital role in determining the toxic effects. Spherical shapes possess a lower surface area and tend to be less toxic than structures with larger surface areas, such as nanosheets. Compared to extracellular integration, the extent of cytotoxicity of photothermal nanomaterials (PNMs) is higher during intracellular integration. After compartmentalization, toxicity can be decreased by controlling the fluidity of PNMs via binding in the designated compartments, such as the cytoplasm. Cytotoxicity of PNMs can be controlled by compartmentalizing them in a periplasmic space. The availability of a wide variety of PNMs enables the selection of specific materials based on their stability and toxicity profiles.

## 3. Inorganic Semiconductor–Microorganism-Based NBHs

Inorganic semiconductor–microorganism-based NBHs represent one of the most promising frontiers in AP research. These systems synergize the light-harvesting prowess of inorganic semiconductors with the metabolic versatility of microorganisms, enabling solar-driven production of fuels and chemicals with unprecedented efficiency. By interfacing semiconducting QDs, metal oxides, or perovskites with bacteria, algae, or fungi, researchers have unlocked pathways to bypass natural photosynthetic bottlenecks and engineer novel biohybrid energy conversion platforms. These systems facilitate direct electron transfer, enabling sustainable solar-to-chemical energy conversion for fuel production.

### 3.1. Cadmium Sulfide–Microorganism-Based NBHs

Colloidal inorganic NMs from group IIB–VIA semiconductors are highly effective visible light absorbers. Among them, CdS stands out as a promising candidate and is widely utilized for fabricating NBHs [26]. Photoelectrons generated on the surface of CdS thermodynamically drive various biological redox reactions, effectively converting light into nutrients. Moreover, the presence of proteins and peptides in cellular environments facilitates the biomimetic and in situ synthesis of nanoscale CdS within microbial cells. CdS QDs have a band gap of approximately 2.4 eV, enabling them to absorb visible light with wavelengths shorter than 517 nm. Additionally, the band gap of CdS can be tuned by adjusting particle sizes, offering significant flexibility. Notably, CdS QDs can harvest visible light up to 800 nm, maximizing the utilization of visible light, which constitutes about 40% of sunlight. CdS QDs have been employed in AP hybrids due to their shorter electron transfer pathways and higher surface area/activity [27,28,29].

**Figure 1:**
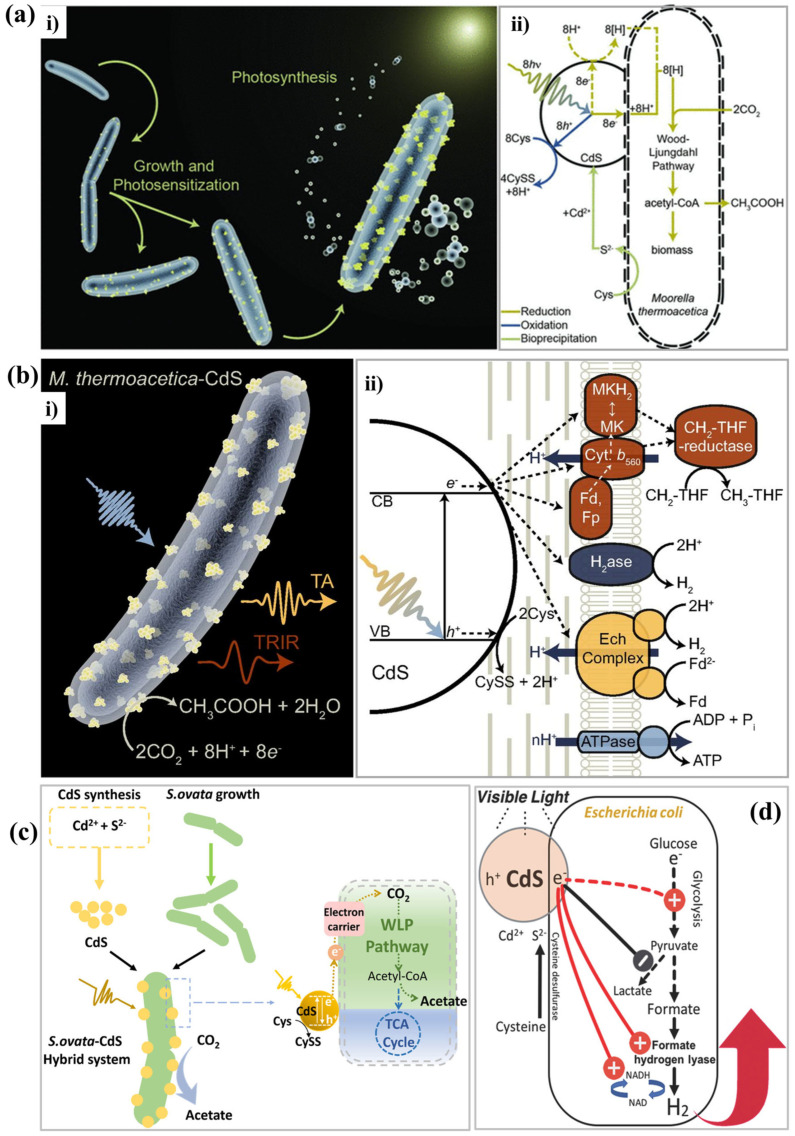
CdS-based NBHs for photochemical solar-to-chemical production. (**a**,**b**) Moorella thermo-acetica-CdS biohybrid system showing (**i**) growth and photosensitization processes with possible reaction pathways (**ii**) for photosynthetic conversion of CO_2_ to acetic acid [30,31]. (**c**) Formation of *S. ovata*−CdS biohybrid system and its CO_2_ reduction under light irradiation [38]. (**d**) Proposed mechanism for enhanced H_2_ production using CdS biohybrids [39]. Figures reproduced with permission from cited references.

M. thermoacetica, a non-photosynthetic bacterium, was coupled with CdS NPs and used for the conversion of CO_2_ to acetic acid [30]. The hybrid system (Figure 1a) was formed by the precipitation of CdS by *M. thermoacetica* upon the addition of Cd^2+^ and Cys as a sulfur source. These biologically precipitated CdS NPs within the *M. thermoacetica* cells function as a light harvester to sustain cellular metabolism. Following day one, the cell count and the viability doubled during photosynthesis. The growth depended on the amount of CdS and nutrients supplied, suggesting that this hybrid system is self-reproducing and sustained under solar energy. Furthermore, this hybrid sustained the selective conversion of CO_2_ to acetic acid in the dark and light cycle, continuously for several days. At the specified Cys concentration, 90% of CO_2_ was converted into acetic acid, while the rest of the CO_2_ was assimilated into biomass. Thus, the light flux/bacterial cell can be tuned by adjusting the concentration of the hybrid. This work rendered a self-replicating strategy for the solar light-assisted CO_2_ reduction with high quantum yield. *M. thermoacetica*-CdS hybrid was expected to expand the utility of natural photosynthesis.

Kornienko et al. further studied this hybrid to gain the mechanistic insights from the reaction involved at the complex biotic and abiotic interface (Figure 1b) by using conventional transient absorption spectroscopy (TAS) [31]. Mild and biocompatible probing conditions were adopted for the TAS. It is well established that photoexcitation of CdS leads to oxidation of Cys to cystine (CySS), where the photogenerated electron is transferred to a membrane protein, and the hole is quenched by H^+^. The rates of photogenerated electron transfer and the quantum efficiency were increased with the increase in the activity of hydrogenase (H_2_ase). According to the TAS study, the conversion of CO_2_ to acetic acid followed a two-pathway mechanism: (i) formation of H_2_ase-mediated H_2_ as a molecular intermediate via a charge-transfer pathway with a high quantum efficiency occurs over a longer duration (24 h); and (ii) formation of acetic acid via an enzymatic energy-transducing pathway occurs over a short duration (3 h). The application of conventional TAS to study the mechanism in a complex nanohybrid established a rational characterization framework for developing next-generation solar energy conversion systems for renewable chemical synthesis.

Ding et al. designed the NBH for the renewable production of chemical and biofuels [32]. The microorganism was attached to the Zinc sulfide (ZnS) shell of the core-shell inorganic semiconductor QD assembly prepared by a layer-by-layer deposition. The core materials were CdS, CdSe, InP, and copper zinc tin sulfide (Cu_2_ZnSnS_4_). Based on the strong light absorption, seven different core materials capable of undergoing excitation by UV to NIR light were used as the core, while two monolayers of ZnS were kept as the shell. The microbes used were *Azotobacter vinelandii (A. vinelandii)* and *C. necator*. These non-photosynthetic bacteria use sugar and biofuels to activate the enzymatic processes, enabling the reduction of renewable chemical feedstocks (CO_2_, H_2_O, and air) into fuels and chemicals. Ding et al. combined these bacteria with the light-absorbing QDs, used for the selective activation of the molybdenum−iron nitrogenase (MFN) in *A. vinelandii*, hydrogenases, and quinones in the Fe−S clusters of *C. necator*. The chemical binding affinity of Zn with specific functional groups in organisms was utilized to couple the QD assembly with the microorganisms. Increasing the thickness of the ZnS shell ensured the site-specific attachment of microbes, improved biocompatibility, and the extent of injection of a photogenerated electron into the active sites in the enzymes. However, high shell thickness increased passivation and acted as a barrier against the charge injection from QD cores. The excitation property of these QD assemblies enabled enzyme activation upon applying light, an electromagnetic stimulus. Light activation of this NBH endowed the conversion of CO_2_, water, and air nitrogen into different chemicals and biofuels. The light-induced reduction of CO_2_ using these NBH exhibited a high turnover number (TON). The TON for the renewable production of biofuels like H_2_, 2,3-butanediol, isopropanol, ethylene, formic acid, ammonia, and methyl ketone (C_11_-C_15_) was ∼10^6^–10^8^ (mols of product per mol of cells). The maximum light-to-chemical conversion efficiency was 16–20%.

Ye et al. constructed an NBH based on a whole-cell microorganism, a methanogen, and a CdS semiconductor for achieving CO_2_ to methane conversion [33]. *M. Barkeri* was used as a model methanogen as its metabolism favors highly efficient conversion of CO_2_ to other chemicals. The CdS NPs with a high absorption coefficient were used as a photosensitizer. The surface electrostatics of CdS NPs support the integration of a methanogen and decrease the charge transfer barrier. The facile interaction between the photoactive CdS NPs and *M. barkeri* resulted in a high methane production rate (0.19 μmol/h) with a quantum efficiency (0.34%) comparable to plants and algae. When exposed to light irradiation, this NBH exhibited a higher electrical conductivity and photocurrent than *M. barkeri* alone. CdS photosensitizer absorbs photons and generates more photo-exited electrons, which are then transferred to *M. barkeri*. However, when used alone, slower electron uptake occurs in *M. barkeri*. Thus, methanogenesis (CO_2_ to CH_4_ conversion) is higher in the case of a hybrid than in M. barkeri alone. In addition, there was a 151.4% increase in the copying of the mcrA gene, confirming the robustness of this NBH. Membrane-bound proteins played a crucial role in the process of photoelectron transfer. The H_2_ases and cytochrome pathways mediated electron–hole separation, facilitating the conversion of CO_2_ to methane. The authors suggested that these findings can be useful for the further exploration of self-replicating solar energy-driven CO_2_ to methane conversion systems.

Kumar et al. combined CdS and electro-active bacteria (EAB) to construct a tandem hybrid for CO_2_ conversion [34]. The EAB strains used were *Acetobacterium woodii (A. woodii)*, *Clostridium ljungdahlii (C. ljungdahlii)*, *M. thermoacetica*, and *Pseudomonas aeruginosa (P. aeruginosa)*. Nano CdS clusters were anchored on the microbial outer cell membrane, facilitating light harvesting and ease of charge transport at the bacterial interfaces. Under visible light irradiation (λ > 400 nm), this hybrid enabled the conversion of CO_2_ into single and multi-carbon compounds. Either Cys or H_2_S was used as a sulfur source. In both cases, the conversion of CO_2_ to acetic acid was dominant. Along with acetic acid, fractions of methanol, ethanol, propanoic acid, butyric acid, and hexanoic acid were also formed. In the period of 5 days, using Cys as a sulfur source resulted in a higher CO_2_ to product conversion (2.4 g/L) than using H_2_S (2.04 g/L). When Cys and H_2_S were used as the sulfur source, the concentration of acetic acid was 1.46 g/L and 1.55 g/L, respectively. The use of H_2_S produced more hexanoic acid and less methanol. In comparison, Cys resulted in a two-fold higher methanol fraction and no hexanoic acid. The ease of light harvesting by this hybrid was found to be a unique feature that supports sustainable solar energy-based conversion of CO_2_ into a variety of other chemicals.

The hybrid system, compatible with complex reaction systems, is required for efficient AP. Such a compatible system can facilitate the production of various chemicals via multiple CO_2_ conversion reactions. To derive the synergistic output from a system combining photocatalysis and biocatalysts, there should be a pathway for transporting reducing equivalents from the photocatalyst to the biocatalyst, and a compatible interface must be provided between them. Although photoelectrochemical cells are suited for this purpose, optimized methods are required to obtain a facile and controllable system. Inspired by natural photosynthesis systems where the chloroplast served as the energy conversion center and employs thylakoids to couple the photo- and bio-reactions, Zhang et al. designed an artificial thylakoid by decorating the inner side of protamine–TiO_2_ (PTi) hybrid microcapsules with CdS QDs [35]. The chloroplast-based natural photosynthesis system can harvest 130 tW of solar energy and can convert 100–120 Gts of carbon into biomass/year. The PTi-CdS microcapsules were prepared using poly(styrenesulfonate)-doped CaCO_3_ microspheres as the template via a coprecipitation method. Through an electrostatic interaction, the sulfate groups (SO_4_^2−^) in the template absorb Cd^2+^ and facilitate the deposition of CdS QDs. Using a SiO_2_ template resulted in larger aggregates instead of QDs. The photobiocatalytic conversion of CO_2_ was performed under visible light (405 ± 5 nm). During the photobiocatalytic conversion reactions, these microcapsules were coupled with either formate dehydrogenase alone or with a combination of formate, formaldehyde, and alcohol dehydrogenases. The photocatalytic oxidation and biocatalytic reduction were compartmentalized in a sequence by using a size-selective capsule wall. Compartmentalization reactions protected the enzymes from ROS-mediated denaturation. The electronic coupling and band structure formed by CdS with PTi favored the separation of electrons and holes. The effective charge carrier separation afforded a higher NADH regeneration rate (4226 ± 121 μmol/gh), and the optimized yield was 93.03 ± 3.84%. With single and multiple enzymes, formate and methanol production were 1500 and 99 μM/h, respectively. This system can be applied to construct a wide range of enzyme-dependent or NADH-free CO_2_ conversion systems.

Wang et al. coated the CdS NPs on the surface of photosynthetic bacterium, *R. palustris* and explored the system for the production of C2+ chemicals via visible light-mediated CO_2_ conversion [36]. This bacterium has been applied in water treatment, chemical and food additive production, and environmental biodegradation. Besides that, it can acquire electrons from external photosensitizers, switch across various metabolic processes, and survive in different environments and cheap energy sources. The CdS was generated by bacterium-mediated precipitation of Cd^2+^ via cysteine desulfurase. Notably, in this system, the hazardous Cd^2+^ was transformed into useful CdS NPs. The transfer of electrons from CdS to bacteria increased the NADPH cofactor, which promoted the formation of a Calvin cycle intermediate, glyceraldehyde-3-phosphate. As a result, it enhanced the production of carotenoids (122%), polyhydroxybutyrate (PHB) (147%), and biomass (148%). In the natural solar/dark cycle, the % of carotenoid, PHB, and biomass production reached 135%, 117%, and 139%, respectively. The maximum photosynthetic efficiency of this NBH was 5.98%. The interface between CdS and bacterial cells promoted the generation and transduction of electrons. Under autotrophic conditions, this NBH system survived well over natural *R. palustris*. This NBH can be expanded to solar-driven advanced CO_2_ conversion applications.

Xu et al. coupled the hydrothermally synthesized hexagonal crystal of CdS nanorods (NRs) with the heterotrophically grown *Cupriavidus necator H16*, a metabolically versatile bacterium capable of subsisting on H_2_ and CO_2_ as its sole sources of energy and carbon [37]. This hybrid was explored for the production of PHB, a bioplastic material. Under light irradiation, the hydrothermally synthesized CdS NRs–*C. necator* H16 combination produced a 1.5-fold higher PHB than the commercial CdS–*C. necator* H16 combination. In 120 h, CdS NRs–*C. necator* H16 converted fructose into PHB (1.41 g), and within 48 h, 28 mg of PHB was obtained from CO_2_ conversion. When tested with other microbes, including heterotrophic *Saccharomyces cerevisiae* (*S. cerevisiae*) 288C, *Gluconacetobacter xylinus*, and *Anabaena* sp. PCC7120, CdS had a negative effect, highlighting its specificity towards *C. necator* H16. The authors suggested that before coupling CdS NRs with microbes, optimization of photocatalysts with the relevant synthesis process is necessary. For high productivity, an optimized balance must be maintained between the concentration of CdS NRs and the number of *C. necator* H16. The extracellular catalase enzymes generated by *C. necator* H16 have been shown to protect it from the oxidative stress caused by CdS. These findings emphasize the need to identify photocatalyst-specific microbial species to achieve high-performance NBHs.

Similar to sulfur-aided CO_2_ reduction by H_2_, an NBH made by attaching an electroautotrophic microorganism, *S. ovata*, to CdS was developed to demonstrate the light-driven conversion of CO_2_ into multi-carbon compounds [38]. The electrons generated by irradiation of CdS were transferred to *S. ovata* and used to reduce CO_2_ to formate and then to acetate, following the Wood−Ljungdahl pathway (Figure 1c). CdS NPs (50 to 250 nm) exhibited a maximum absorbance at 400 nm, and the presence of *S. ovata* had no remarkable impact. The optimal concentration of CdS, 1.00 mM, was found to be safe for *S. ovata*. Additional photovoltaic devices were not used for the photo-energy conversions. The active duration time and the quantum yield of the system were 5 days and 16.8 ± 9%, respectively. The key enzymes involved in this CO_2_ conversion were flavoprotein, ferredoxin, rubrerythrin, formate-tetrahydrofolate ligase, methyltransferase, thioredoxin, and 5-methyltetrahydrofolate: corrinoid Fe−S protein. In the nano-biohybrids, these enzymes were up-regulated. It was suggested that these findings could be useful for designing genetically engineered microbes for solar energy harvesting.

Under anaerobic conditions, endogenous [Ni–Fe]-hydrogenase induces the H_2_ production. When subjected to stimulated conditions, *E. coli* precipitates CdS. Wang et al. prepared *E. coli*-CdS NBH by precipitating CdS on the surface of *E. coli* and utilized it for visible light-driven H_2_ production [39]. *E. coli* anaerobically synthesizes endogenous [Ni-Fe]-hydrogenase capable of producing H_2_ (Figure 1d). Thus, additional genetic engineering procedures are required to induce exogenous hydrogenase into the host organism. This work seems to be the first effort focused on the direct transfer of electrons generated on the photosensitizer deposited on the extracellular side to intracellular biological processes. This NBH increased the H_2_ production by 30% (400 μmol) after 3 h. The apparent quantum efficiency was 7.93% and 9.59% under visible light sources having the wavelengths of 470 and 620 nm, respectively. The observed quantum efficiency was higher than that of many photoheterotrophic bacteria. The interaction between photogenerated electrons and *E. coli* cells was confirmed. Under natural sunlight, within 3 h, this hybrid system generated an additional 120 μmol H_2_ from the wastewater. Utilizing wastewater and natural sunlight to convert toxic Cd^2+^ into useful CdS makes this NBH more significant in both environmental and practical aspects. However, O_2_ intolerance of hydrogenases and the low biocompatibility of synthetic photosensitizers limit the use of whole-cell hybrid systems for H_2_ production.

To achieve effective biomethanation under visible light, Chen et al. developed an NHB combining CdS QDs and *R. palustris*, a genetically engineered bacterium with significant potential for solar-powered CO_2_ conversion [40]. The CdS QDs were anchored on the surface of *R. palustris* cells, enhancing visible light harvesting. Compared to *R. palustris* alone, this NBH exhibited higher cell viability, negligible cell damage, and achieved a biomethanation performance of ~79%. The cell density and CdS QDs concentrations are critical parameters, as light intensity and electron–hole scavengers influence the extent of biomethanation. By optimizing these process parameters, a high methane production of 171 nmol/mg total protein was achieved under visible light (400–500 nm). This work provided an effective route to photo-driven biomethanation under unfavorable light conditions.

Compared to other AP systems, the periplasmic photosynthetic system decreases the distance between the outer cell membrane and the light-absorbing NMs. Therefore, this NBH offers a quicker and shorter electron transfer pathway. The existence of space between the inner and outer cell membrane (periplasmic space) has also been shown to reduce the cytotoxicity of NPs. Thus, periplasmic NBH is expected to deliver sustained chemical or energy production. To improve the electron transfer efficiency in periplasmic photosynthetic hybrids, Liang et al. developed *E. coli*-CdS hybrids by engineering the fluidity of the cell membrane and periplasmic cytochrome network [41]. A two-step synthesis process was followed to obtain CdS NPs within the periplasmic space of wild-type *E. coli* (MG1655). The localization of specific genes involved in the synthesis of Cys increased the amount of Cd^2+^ ions in the periplasmic space. Similarly, the Cys desulfhydrase from *Treponema denticola* was targeted to generate the ME-1 strain to catalyze the conversion of Cys to H_2_S. The reaction between H_2_S and Cd^2+^ ions rendered the CdS NPs. Cys was used as a sulfur source for CdS and as the sacrificial electron donor. This avoided the use of an external sacrificial reagent. Improvements were observed with the utilization of reducing equivalents and NADH generation. These factors led to a significant increase in succinate production (121.8 g/L in 5 L fermenters). This system established a microbial consortium using glucose capable of delivering consistent production of electric energy (power density of 225.3 mW/m^2^ in 17 days). These findings advanced the industrial process aimed at developing a microbial consortium offering long-term and sustainable production of chemicals and electrical energy.

To overcome the limitations of enzymes and synthetic catalysts, Wei et al. combined semiconductors, enzymes, and whole bacterial cells for H_2_ production via self-photosynthesis [42]. The hybrid was formed by integrating CdS NPs capable of binding with proteins, oxygen-tolerant [NiFe]-hydrogenase, and biomimetic silicon-encapsulated engineered *E. coli* cells. CdS NPs were bound selectively to the Pb^2+^ responsive regulatory protein on the *E. coli* cell membrane. This led to the precipitation of CdS on the outer membrane of *E. coli* via biosynthesis using Cys residues as the sulfur source. Even at natural aerobic conditions, this photo-biocatalyst combination enabled H_2_ production for 96 h.

Single-cell level mechanistic studies can provide quantitative guidance towards strategies aiming to increase the efficiency of NBHs. Based on label-free structural color spectroscopy, Gao et al. conducted an operando study to evaluate various strategies used to overcome the sluggish light-harvesting behavior in NBHs [43]. They studied the dynamics of photosynthetic H_2_ generation in a single-cell *E. coli*-CdS hybrid, where 30 nm CdS NPs were distributed on the cell membrane. Light illumination induced blue shifts, indicating the accumulation of H_2_ produced by biogenic semiconductors on the microbial cell membrane. By quantifying the spectral shift in the structural color scattering, the authors observed the sunshade effect caused by the H_2_ bubbles, accumulated on the outer membrane of the microbial cells. This intrinsic sunshade constraint inhibited the light-harvesting efficiency of photosynthetic NBHs. Incorporation of a tension eliminator, cetyl(trimethyl ammonium bromide), into the hybrid effectively surmounted the bubble sunshade effect and facilitated a 4.5-fold increase in the light-harvesting efficiency. Findings of this study on the transmembrane transport of gas products can be useful for optimizing photosynthetic NBHs for efficient light-harvesting.

In the CdS-based NBHs discussed above, CdS nanomaterials were either adhered to the cell surface or localized within the cellular membrane. Integration of CdS nanomaterials with microorganisms was achieved through both external synthesis and in situ precipitation of Cd^2^⁺ ions within microbial cellular compartments. General synthesis techniques used to externally produce CdS nanomaterials include hydrothermal synthesis, chemical deposition, in situ precipitation, wet chemical synthesis, electrostatic deposition, and sol-gel methods. The use of such general methods offers advantages for scalability. Precipitation of Cd^2^⁺ in the periplasmic space compartmentalized the CdS nanomaterials in that region, forming periplasmic-type extracellular hybrids. Externally synthesized CdS quantum dots (QDs), nanorods (NRs), and nanoparticles (NPs) appear to be impermeable to the cell membrane, and thus primarily yield extracellular NBHs. The particle size of CdS nanomaterials typically ranged from 5 to 20 nm. Therefore, developing strategies to achieve intracellular localization of CdS within microbial cells remains an important research priority.

Integration of CdS/ZnS (core–shell) QDs and MFN in *A. vinelandii*, as well as Fe–S clusters with *C. necator*, has demonstrated a light-to-chemical conversion efficiency of 16–20% [32]. The quantum yield for CO_2_-to-methanol conversion using CdS NPs in combination with *M. barkeri* reached 0.34%, which is comparable to natural methanogenesis in plants and algae [33]. For hydrogen production from wastewater, *E. coli*-CdS NBHs, prepared via in situ precipitation of Cd^2^⁺ to localize CdS NPs, exhibited apparent quantum efficiencies of 7.93% and 9.59% under illumination at 470 nm and 620 nm, respectively [39]. Using wastewater as a feedstock offers an economically viable route for hydrogen production.

CdS QDs and engineered CdS surfaces demonstrated a compatible nano-bio interface and scalability potential. However, the complex and often costly synthesis of nanomaterials remains a barrier to practical implementation. Despite exhibiting strong visible light absorption and moderate-to-good electron transfer efficiency, challenges such as surface passivation, photocorrosion, and precise control over the nano-bio interface persist, particularly in systems employing nanoparticles.

### 3.2. Titanium Dioxide and Silicon Nanowires–Microorganism-Based NBHs

TiO_2_ is a stable, competent, thoroughly studied photocatalyst that works under UV light. Despite the development of various photocatalysts, the low cost and biocompatibility of TiO_2_ nanomaterials sustain them as a potential option for energy conversion applications such as solar fuel production and CO_2_ conversion. In AP hybrids, TiO_2_ is either used alone as a light-absorbing unit in NBHs bearing enzymes and microbial cells or as a passivation layer for other PNMs such as Si, CdS, and CdTe. Silicon nanowires (Si NWs) have advantages over TiO_2_. Si NWs effectively capture visible or solar light and are used for visible or sunlight-driven H_2_ and CO_2_ conversion [44,45,46].

**Figure 2:**
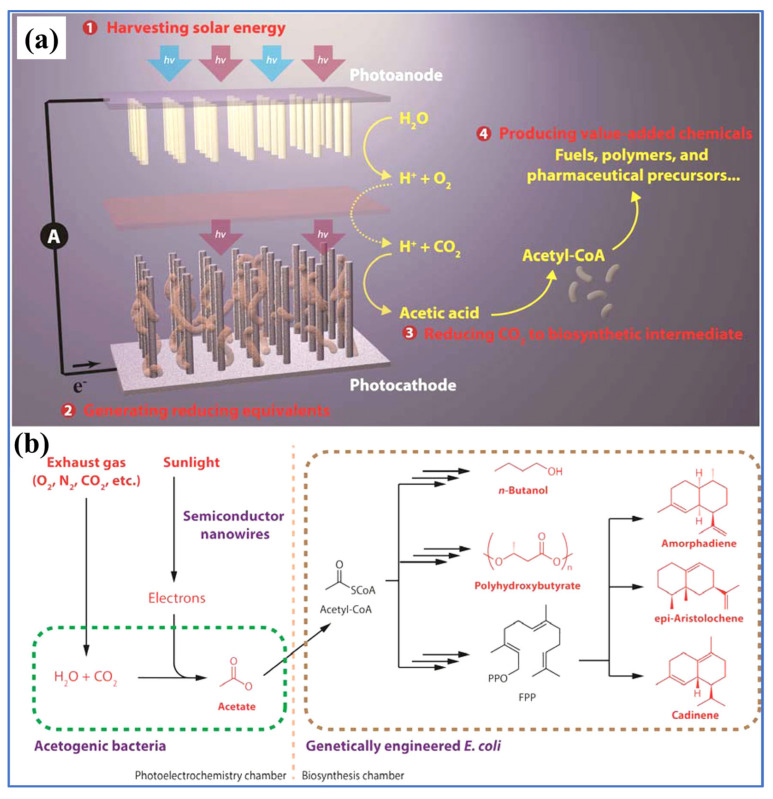
TiO_2_- and Si-based NBHs for a general artificial photosynthetic approach. Proposed technique for solar-powered CO_2_ fixation and solar energy conversion into (**a**) biosynthetic intermediates and other value-added products, and photoelectrochemical conversion (**b**) that demonstrates under mild conditions to directly treat and generate biosynthetic intermediates, and its upgraded conversion into liquid fuels, biopolymers, and pharmaceutical precursors. FPP = farnesyl pyrophosphate [47]. Figures reproduced with permission from cited references.

Liu et al. demonstrated the first example of photo-electrosynthesis of value-added chemicals (fuels, polymers, and pharmaceutical precursors) using semiconductor nanowires (NWs)–bacteria hybrids [47]. They interfaced an array of semiconducting Si and TiO_2_ NWs with the CO_2_-reducing bacteria, *S. ovata* (Figure 2). This system reduced the CO_2_ under mild reaction conditions involving an aerobic atmosphere, temperature below 30 °C, and neutral pH. The NWs array possesses advantages such as oxygen tolerance and high CO_2_ fixation activity. The NWs arrays absorbed solar energy and transferred the reduced equivalents to the bacteria. Using this NBH, photosynthetic production of acetic acid was realized under aerobic conditions (21% O_2_). Under stimulated sunlight, acetate production was continued up to 200 h with 0.38% solar energy conversion efficiency. Using the genetically engineered *E. coli*, acetate can be activated to form acetyl-coenzyme and used as a building block for synthesizing n-butanol, PHB, and natural isoprenoid products.

**Figure 3:**
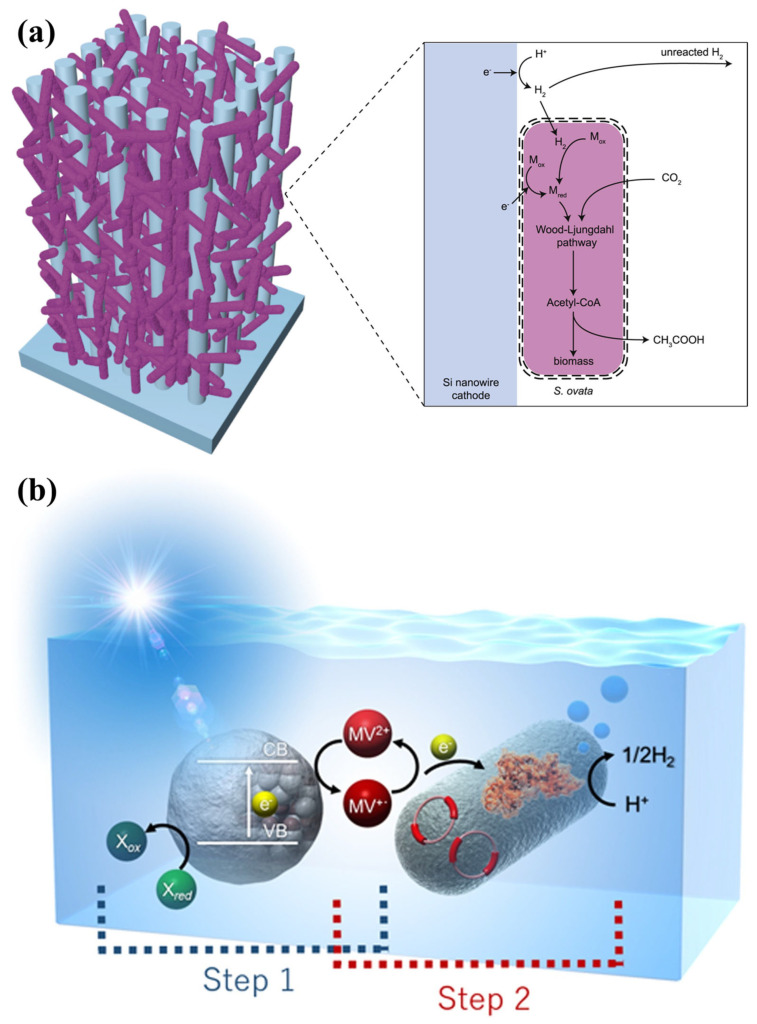
(**a**) Close-packed Si nanowire–bacteria hybrid system (left) and its reaction pathway (right) showing electron transfer process from Si nanowire cathode to S. ovata to generate the intracellular reducing equivalents, which eventually become converted into acetate and biomass (Wood–Ljungdahl pathway) [48]. (**b**) Stepwise photocatalytic H_2_ production using a multicomponent biohybrid system (inorganic semiconductor, an electron mediator, and a whole-cell biocatalyst (recombinant *E. coli*). Initially, methylviologen (MV) reduction occurs by an inorganic semiconductor, followed by H_2_ production by a whole-cell biocatalyst from reduced MV [49]. Figures reproduced with permission from cited references.

Su et al. directly interfaced CO_2_-reducing *S. ovata* with a conductive p-type Si NWs array (cathode material) (Figure 3a) [48]. Inorganic phosphate buffer was used as an electrolyte medium (initial pH = 7.2). In the electrochemical cell, *S. ovata* suspension was inoculated in the cathodic chamber. The average cell distribution per NW was 2.6. The problem of poor bacteria–NMs interface was mitigated by tuning the pH of the bulk electrolyte and increasing the buffering capacity. These changes led to the formation of a close-packed cathode, NBH of NW, and bacteria. This close-packed NBH was operated in a CO_2_ atmosphere, where the reducing current density was 0.65 ± 0.11 mA/cm^2^ (at ~1.2 V vs. SHE). When integrated with a photovoltaic device, over 7 days, this NBH demonstrated the solar-powered CO_2_ to acetate conversion efficiency of 3.6% and the faradic efficiency of 80%. A robust interface between the microbe and the cathode interface resulted in a high CO_2_-reducing current density of 0.65 mA/cm^2^. Collectively, these insights gained from the systematic investigation of bioinorganic interfaces with varying operation parameters can be used to increase the rate of CO_2_ reduction.

An inorganic NBH system was formed by combining anatase TiO_2_, methyl viologen (MV) (electron mediator), and a whole-cell catalyst containing an enzyme-microbe. The whole-cell catalyst was formed by [FeFe]-hydrogenase and maturase gene-harboring *E. coli* and explored for H_2_ production (Figure 3b) [49]. Under a 300 nm light source, the apparent quantum yield of H_2_ production was 0.3%. First, MV was reduced by TiO_2_, resulting in H_2_ production in conjunction with reduced MV and biocatalyst. The MV reduction process was investigated in detail. The rate of TiO_2_-induced MV reduction was increased by 300 times upon adding 100 mM Tris-HCl (pH 7), 150 mM NaCl, and 5% (*v*/*v*) glycerol, compared to using 100 mM ascorbate with TiO_2_. With enhanced MV reduction, the 300 and 350 nm light increased the apparent quantum efficiency to 60.8% and 52.2%, and H_2_ production to 26.4% and 31.2%, respectively. This combined photocatalyst and whole-cell biocatalyst provided noble metal-free, efficient, and cleaner H_2_ production.

Microbial CO_2_ fixation under photoelectrochemical conditions is one of the promising sustainable fuel production methods. Chen et al. fused the palladium (Pd, 2.5 nm)- coated porous TiO_2_ NPs with bacteriorhodopsin, halobacterium purple membrane-derived vesicles (PMVs) [50]. The purple membrane covers ~80% of the cell membrane of the *archaea Halobacterium salinarum*. Bacteriorhodopsin acts as a light-mediated proton pump. Incubation of PMVs with Pd-coated TiO_2_ NPs rendered a core-cell assembly mimicking the cell and used for performing visible light-driven CO_2_ reduction. Bacteriorhodopsin acted as a photosensitizer and retained its proton-pumping function. Photogenerated electrons were injected into the conduction band of TiO_2_ NPs. The simultaneous electron trapping by Pd and accumulation of protons in the cytomimetic architecture rendered proton-coupled multielectron transfer and reduced CO_2_. The bioinorganic interface between microbes and the cathode plays a crucial role in determining the efficiency. Due to the poor bacteria–NMs interface and harsh alkaline environment, the rate of bioelectrochemical CO_2_ reduction was limited.

Integration of a solid cathode with reducing bacteria is a promising strategy for the sustainable production of fuels via CO_2_ conversion. Kim et al. designed a robust NBH comprising methanol-adapted *S. ovata* as a biocatalyst and an array of semiconducting Si NWs [51]. Autotrophic metabolic activity of *S. ovata* was enhanced under methanol and H_2_/CO_2_. Thus, methanol adaptation boosted the CO_2_-reducing current density of *S. ovata* on the Si NWs cathode. Compared to wild-type *S. ovata* strains, Si NWs and methanol-adapted *S. ovata* whole-cell biohybrids decreased the interfacial charge transfer resistance and accelerated the charge transfer from the cathode to bacteria. Due to the synergistic effect of the high surface area of Si NWs and catalytic activity of methanol-adapted *S. ovata* stains, the extent of CO_2_ reduction was enhanced with a superior faradaic efficiency (of acetate) reaching 100%, and 2.4-fold greater current density (0.88 ± 0.11 mA/cm^2^) than the wild strains. At the optimized conditions (biocatalyst loading, applied overpotential, and electrolyte pH), this system exhibited a 4.4 times greater CO_2_-reduction than the typical whole-cell catalyst based on methanol-adapted *S. ovata*.

Similar to CdS-based NBHs, the integration of silicon nanowires (Si NWs) and titanium dioxide nanoparticles (TiO_2_ NPs) has enabled the construction of extracellular hybrid systems with enhanced CO_2_ conversion capabilities. NBHs developed by integrating Si NWs and TiO_2_ NPs with *S. ovata* demonstrated the conversion of CO_2_ into acetic acid under solar irradiation, achieving a solar energy conversion efficiency of 0.38% [47]. Notably, when p-type Si NWs were integrated with *S. ovata* in combination with a photovoltaic device, the CO_2_-to-acetate conversion efficiency increased significantly to 3.6% [48].

In another approach, the combination of TiO_2_, methyl viologen (MV), and genetically engineered *E. coli* harboring [FeFe]-hydrogenase and maturase genes has shown promise for hydrogen (H_2_) production. The apparent quantum efficiencies under 300 nm and 350 nm light sources were 60.8% and 52.2%, respectively [49]. The robust and inexpensive TiO_2_ NPs and MV, along with their compatibility with *E. coli*, make this NBH system particularly promising. However, prolonged exposure to high-energy light sources can damage *E. coli* cells and reduce overall system efficiency. The reliance of TiO_2_ on UV light (~300 nm) significantly limits its ability to utilize the full solar spectrum.

Furthermore, the integration of palladium-coated porous TiO_2_ NPs with bacteriorhodopsin resulted in a visible light-active NBH system. However, its CO_2_ conversion capability remained limited due to poor interfacial contact and low stability under alkaline conditions [50]. Additionally, studies have shown that integrating arrays of Si NWs with *S. ovata* enhanced acetate production by 4.4-fold compared to *S. ovata* alone [51].

## 4. Other Inorganic Nanomaterial/Semiconductor–Microorganism Hybrids

Despite the widely studied CdS, TiO_2_, and MoS_2_ semiconductors, other inorganic semiconducting QDs (e.g., InP, CdSe), and other nanomaterials such as iron oxide (Fe_2_O_3_), gold (Au), and CoP NPs, have been explored as photocatalysts in AP hybrids. These inorganic nanomaterial/semiconductor–microorganism-based NBHs have been designed to improve efficiency, stability, and scalability in AP systems. These alternative materials offer unique optical, electronic, and catalytic properties that enhance microbial interactions for sustainable energy production. The band gap of InP (1.34 eV) is smaller than CdS (2.4 eV), absorbs visible light, is biocompatible, resistant to oxidation, and can be integrated with microbial cells. InP QDs can form intracellular NBHs, and generated photoelectrons can directly participate in the regeneration of cytoplasmic bioactive cofactors [52]. Monodisperse CdSe QDs are water-soluble and are used as efficient visible light photocatalysts with a tunable band gap. Highly luminescent CdSe QDs are also widely utilized in biological imaging and sunlight harvesting [53,54]. CoP is a sustainable and efficient catalyst derived from earth-abundant cobalt and phosphate [55]. Au NCs and Au NPs are environmentally benign materials capable of permeating the cell membrane to form intracellular NBHs like InP QDs [56]. Furthermore, chalcogenides such as silver indium disulfide (AgInS_2_), copper indium disulfide (CuInS_2_), indium (III) sulfide (In_2_S_3_), and zinc sulfide (ZnS) are also used in AP hybrids. These chalcogenides are narrow band gap semiconductor photocatalysts with visible light absorption properties, and their high active surface area facilitates the formation of AP hybrids with enhanced quantum efficiency [57].

**Figure 4:**
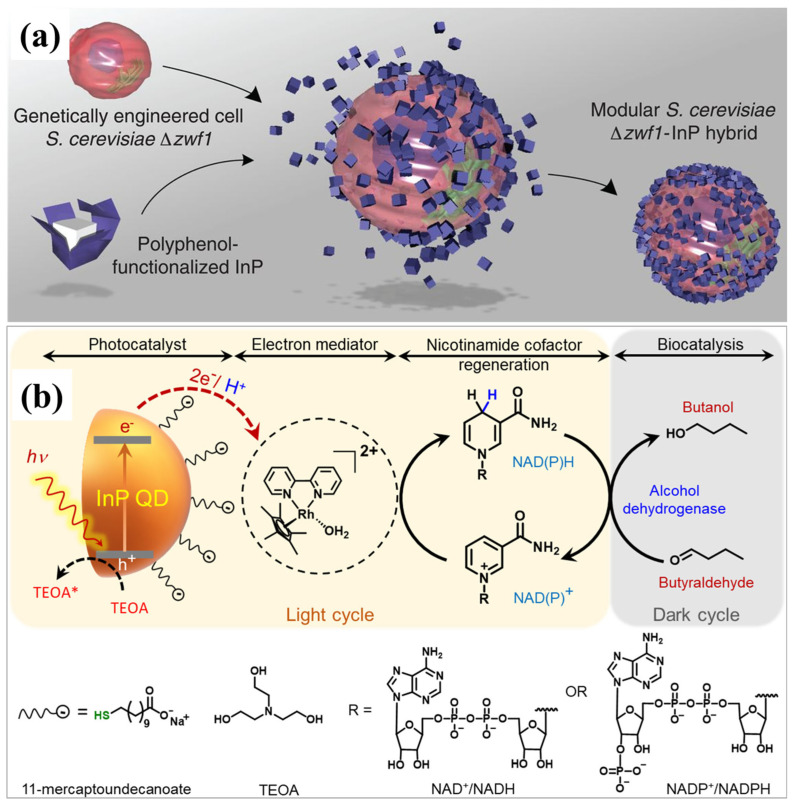
Inorganic PNMs-based NBHs assembly for engineering artificial photosynthetic systems. (**a**) Polyphenol functionalized InP nanoparticles (NPs) and their assembly on the surface of genetically engineered yeast (S. cerevisiae Δzwf1) [58]. (**b**) Engineering the assembly of multicomponent-based eco-friendly InP QDs hybrids for biocatalytic transformations occurring in the integrated light and dark cycles [59]. Figures reproduced with permission from cited references.

Guo et al. coupled InP NPs with *S. cerevisiae*, a genetically engineered workhorse yeast (Figure 4a) [58]. The direct band gap of InP (1.34 eV) enables the effective utilization of sunlight. InP NPs are biocompatible, stable in an oxygen atmosphere, and accept electrons from various species. These InP NPs were prepared separately and integrated with this yeast using self-assembly methods based on polyphenols. The photogenerated electrons from the InP NPs were transferred to the yeast and used for the regeneration of redox cofactors. This NBH was used for the regeneration of NADPH, which plays a crucial role in the biosynthesis of shikimic acid (SA) and other precursors used in the synthesis of drugs and fine chemicals. SA is a precursor for aromatic amino acids. The highest SA production was achieved after 72 h of exposure to sunlight (5.6 mW/cm^2^). This led to the aerobic growth of the yeast. The yeast cell wall-bound InP NPs contributed to the extracellular transport of photogenerated electrons via a hopping mechanism. The light-irradiated hybrid exhibited SA production amounts of 48.5 ± 2.1 mg/L, 24-fold higher than the NBH under dark conditions, and 11-fold higher than the NBHs unattached InP NPs. The insights gained from this NBH can be used for the rational design of complex biomanufacturing processes.

In natural photosynthesis, nicotinamide cofactors (NAD) play the crucial role in the synthesis of biomass, where NAD shuttles between the dark and light cycles. Chakraborty et al. tried to replicate this process using the NBH combining InP QDs and alcohol dehydrogenase (ADH) and explored the continuous photosynthetic production of butanol (Figure 4b) [59]. InP QDs, [Cp*Rh(bpy)-(H_2_O)]^2+^ complex, NAD redox cofactors [NAD(P)+− NAD(P)H], and ADH, were used as the light-harvesting photocatalyst, electron mediator, redox shuttling moiety, and oxidoreductase enzyme, respectively. This NBH was used to meet the demand of fast photogeneration under light and the integration of NAD in the dark. There was a strong electrostatic interaction between InP QDs and electron mediators. This enabled a fast charge extraction and utilization, which resulted in the selective photogeneration of NAD. The photogenerated NAD triggered the ADH and facilitated the synthesis of butanol via simultaneous and sequential dark/light cycles. Within 30 min, 99% photo-regeneration of NAD cofactors was achieved, and the turnover frequency was ∼1333/h. Due to the constant consumption and regeneration of NAD, the quantity of butanol production was greater than the stoichiometric limit. Butanol formation mimicked the natural thylakoid–stroma system. Notably, InP QDs also proved to be useful for generating NAD cofactors under natural sunlight. Integration of InP QDs photocatalysts and enzymes has been proved to be effective in facilitating the electron shuttling, which is similar to natural photosynthesis. This strategy opens up new possibilities for combining other oxidoreductase enzymes and InP QDs that are useful for the continuous synthesis of a variety of chemicals.

Liu et al. prepared a biocompatible NBH-based biosynthetic system capable of water splitting [60]. An inorganic CoP was used as a catalyst to split water into H_2_ and O_2_ at a lower applied voltage (1.8 to 2.0 V). CoP is an earth-abundant material, resistant to ROS, and safer to bacteria. CoP was coupled with the *Ralstonia eutropha (R. eutropha)*. H_2_ generated by water splitting was consumed by *R. eutropha*, and in the presence of O_2_, CO_2_ was converted into biomass, fuels, and other chemical products. The CO_2_ conversion efficiency was ~50%, together with bacterial biomass and fusel alcohol production. The scrubbing of CO_2_ amounted to 180 g/kWh. When integrated with a solar-to-electric conversion system, high solar-to-chemical conversion was achieved. Integration of this NBH with a typical photovoltaic system resulted in a higher CO_2_ reduction efficiency than natural photosynthesis. The efficiency noticed in the case of photovoltaic devices, biomass, bioplastic, and fuel alcohol was 18%, 9.7%, 7.6%, and 7.1%, respectively. This NBH circumvented biotoxicity, allowed the interfacing of water splitting catalysts with synthetic organisms, and was useful for the distributed production of chemicals via solar energy conversion.

The biocompatibility and the light absorption properties of Au NCs were coupled with the catalytic activity of non-photosynthetic bacteria [61]. Au NCs were employed to address issues related to electron transfer. They were utilized in both in vivo and in vitro cell imaging. *M. thermoacetica,* a whole-cell microorganism, was coupled with Au NCs. This NBH enabled the conversion of CO_2_ to acetic acid. The properties of Au NCs can be tuned due to their discrete energy states, unique geometry, tunable core size, and surface ligands. These features allow the manipulation of electronic structure, cellular uptake, biocompatibility, cytotoxicity, and molecular electronic structures. Translocation of Au NCs into the *M. thermoacetica*, an intercellular photosensitizer, enabled the occurrence of photosynthesis. The energy loss across the cell membrane and sluggish kinetics were circumvented by the transfer of photogenerated electrons from Au NCs to cytoplasmic mediators. Intracellular energy transfer resulted in high cell viability due to the ROS scavenging action by Au NCs and enabled the continuous production of acetic acid for 7 days.

The efficiency of solar-driven CO_2_ to chemical conversion relies on the ability of photosensitizers to utilize broader light irradiation and the specific biocatalytic efficiency of microorganisms. Similarly, for the biological production of H_2_, a tandem inorganic–microorganism hybrid was made by combining AglnS_2_/In_2_S_3_ and *E. coli*, a facultative anaerobic bacterium [62]. In_2_S_3_ NPs were anchored on the surface of *E. coli*, which enabled sulfide precipitation upon the addition of In^3+^ and Cys. Then, to establish the AglnS_2_/In_2_S_3_ junction, AglnS_2_ NPs were immobilized on the In_2_S_3_ via an ion exchange. This hybrid harvests light energy and endogenously synthesizes [Ni-Fe]-hydrogenase in the microbial cell. AglnS_2_/In_2_S_3_ junction enabled greater light absorption and afforded rapid electrical conduction. The photoexcited electrons from the conduction band of In_2_S_3_ transferred to the valence band of AglnS_2_ and used to oxidize Cys to CySS. Direct transfer of photoexcited electrons from AglnS_2_/In_2_S_3_ to genetically unmodified *E. coli* promoted the activity of [Ni-Fe]-hydrogenase and enhanced H_2_ evolution. When exposed to light (λ = 720 nm), a 3.3% quantum efficiency (QE) was achieved during H_2_ production, surpassing the performance of typical photoheterotrophic bacteria. This strategy can be used to interface other metal sulfide hybrids as solar light harvesters in biohybrid systems.

**Figure 5:**
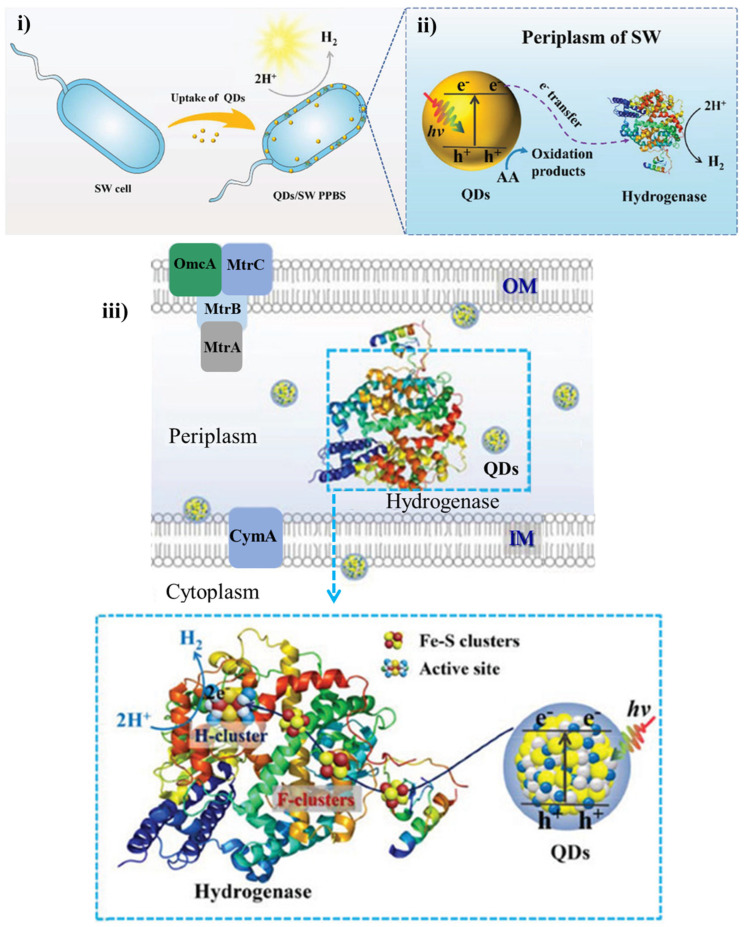
Translocating CuInS_2_/ZnS QDs into the *S. oneidensis* MR-1 (SW) cells (**i**) and its interaction with possible electron transfer routes for solar hydrogen production (**ii**,**iii**) [63]. Figures reproduced with permission from the cited reference.

Sluggish transmembrane electron diffusion severely limits the photocatalytic activity of the inorganic catalyst–biohybrid. To address this issue, Luo et al. constructed a periplasmic photosensitized hybrid (PPH) for solar driven H_2_ production, using CuInS_2_/ZnS QDs (4–10 nm, Eg = 0.6 V) translocated within the *Shewanella oneidensis (S. oneidensis)* MR-1 (SW) cells expressing periplasmic hydrogenases (Figure 5) [63]. The periplasmic space in Gram-negative bacterial cells, situated between the outer membrane and the cytoplasm, enhances favorable light absorption and increases hydrogenase concentration, promoting efficient interactions between hydrogenases and photosensitizers. This proximity to the outer membrane facilitates advantageous biochemical interactions. To realize this, biocompatible CuInS_2_/ZnS QDs were utilized. These QDs are water soluble, resistant to photodamage, highly biocompatible, and possess excellent light-harvesting properties. Identical cell viability was observed in both plain and media with rich QD content. Both photoexcitation and electron transfer occurred in the periplasm of SW cells. Shorter pathway minimizes the energy loss during the transmembrane electron transfer. Minimization of energy loss increased the visible light-driven H_2_ production by eight times compared to bare QDs. The H_2_ production was sustained for 45 h, indicating good viability and stability higher than those of typical hybrids. Gene mutation studies confirmed the transfer of photoexcited electrons into periplasmic hydrogenases and effectively catalyzed H_2_ production higher than QDs-based extracellular photosensitized hybrids. This work provided useful insights for developing an H_2_-generating whole-cell hybrid system with well-balanced light absorption and electron transfer properties.

Direct water splitting by photocatalysis is a thermodynamically challenging process, necessitating a higher concentration of oxidized electron donors. To address this limitation, sacrificial electron donors are expected to be useful for advancing the production of H_2_. Edwards et al. used an entirely different strategy for preparing NBH that is useful in solar-driven H_2_ production [64]. Light-driven catalytic reaction was directly fueled by extracellular electron transfer. Instead of transferring electrons from a semiconducting photosensitizer to a microorganism, the electron transfer was made vice versa (microorganism to semiconductor). This improved the photocatalytic efficiency of nanocrystalline CdSe, which was previously hindered by oxidative reactions. *S. oneidensis* MR-1 was combined with water-soluble CdSe (2.6 nm in diameter), enabling H_2_ production under visible light (530 nm) without the application of an external potential. H_2_ production was sustained for 168 h, and the hybrid system continued for a week by replenishing the bacterial growth medium. Conversely, bare CdSe QDs resulted in negligible H_2_ production due to the absence of electron donors in the modified minimal medium (MM) used for MR-1 culture. In the hybrid, the catalytic reaction was not governed by microbial metabolism, but rather by CdSe QDs. Additionally, the NBH system overcame issues such as photocatalysis-induced oxidative damage, biocompatibility, and the electron transfer process. *S. oneidensis* used lactate in the growth medium as a primary carbon source nutrient to sustain and release respiratory electrons to CdSe QDs, catalyzing H_2_ production. This strategy can be used as a foundation for extending to other NBHs.

Wang et al. sought to clarify the mechanism of the synergistic improvement in H_2_ production from inorganic–microbial NBHs [65]. They developed an NBH containing an intracellular photosensitizer by allowing Au NPs to diffuse inside *Clostridium butyricum cells.* Au NPs aided the biological imaging of mammalian cells due to their biocompatibility. These Au NPs were explored as an intracellular photosensitizer for non-photosynthetic bacteria and were used to convert CO_2_ to acetic acid. This hybrid system enabled H_2_ production under visible light with the apparent quantum yield of 19.31%, which was 88.74% greater than that of the control (dark-fermented *Clostridium butyricum*). The hybrids exhibited a two-fold increase in the expression of enzymes related to H_2_ production (hydrogenase and pyruvate formate lyase) compared to the dark-fermented systems, leading to enhanced H_2_ production. Regardless of pyruvate decomposition, the transfer of photoelectrons to hydrogenase occurred via electron transfer flavoprotein and FAD_2_. Furthermore, there was significant up-regulation of genes related to electron transformation, such as riboflavin synthase, electron transfer flavoprotein, and FAD-dependent oxidoreductase, in the hybrid system. This approach prevented energy consumption caused by the shuttling of photogenerated electrons through the cell membrane. The theoretical findings from this study can be useful for constructing light-driven microbial H_2_ production systems.

The reports discussed above provide insights into NBHs constructed using various inorganic semiconductors. Colloidally synthesized InP quantum dots (QDs) were integrated extracellularly with *S. cerevisiae*, resulting in a hybrid system that exhibited an 11-fold enhancement in CO_2_-to-shikimic acid conversion under light irradiation compared to dark conditions [58]. This highlights the potential of InP QDs–yeast hybrids as rational platforms for complex biosynthetic pathways. Moreover, InP QDs paired with alcohol dehydrogenase (ADH) enzymes yielded a more efficient system than the yeast-based counterpart, enabling continuous butanol production and opening avenues for the synthesis of a broader range of chemical products [59].

Another noteworthy example involves CoP, which formed an extracellular hybrid with *R. eutropha* to catalyze water splitting into H_2_ and O_2_. The H_2_ served as a clean energy source, while O_2_ supported CO_2_ fixation into biomass and chemicals. The earth-abundant cobalt and phosphorus sources, capacity to realize water splitting at a relatively low voltage (1.8–2.0 V), and integration with a photovoltaic module further enhance the system’s capacity for CO_2_ conversion, making CoP based hybrid a cost-effective and scalable platform [60].

In contrast, *M. thermoacetica* was used for in situ biosynthesis of intracellular AuNCs, resulting in a hybrid capable of facilitating intracellular energy transfer. The ROS-scavenging ability of AuNCs contributed to high cell viability and enabled stable CO_2_-to-acetate conversion over one week [61]. Similarly, Au NPs can also penetrate *C. butyricum* cells, forming intracellular hybrids that operate under low-energy light (720 nm) [65].

Integration of *E. coli* with a tandem AgInS_2_/In_2_S_3_ heterostructure resulted in an extracellular system with a notable quantum efficiency of 3.3% for H_2_ production, outperforming native bacteria [62]. Additionally, *S. oneidensis* was integrated with CuInS_2_/ZnS QDs (forming periplasmic hybrids) and CdSe QDs (leading to extracellular hybrids), both demonstrating enhanced solar-driven H_2_ production [63,64].

While these NBHs demonstrate promising functionality across light-driven biosynthetic and energy applications, the practical application faces several challenges. AP Systems incorporating Au, InP, CdSe, and CuInS_2_ nanomaterials must address the issues related to material toxicity, long-term stability, scalability, and economics.

## 5. Carbon-Based Nanomaterials–Microorganism Hybrids

Carbon-based nanomaterials, such as graphene, graphitic carbon nitride (g-C_3_N_4_), carbon nanotubes (CNTs), fullerenes, and carbon dots, have gained significant attention in AP due to their exceptional electrical conductivity, high surface area, and stability. Integrating such carbon-based PNMs with microorganisms can enhance charge transfer, promote photocatalysis, and improve the overall efficiency of solar-to-chemical energy conversion. The g-C_3_N_4_ is an eco-friendly layered metal-free and sustainable photocatalyst with a band gap of 2.7 eV. Melamine, urea, and thiourea are the precursors for preparing g-C_3_N_4_ via pyrolysis. It possesses substantial chemical and thermal stability, works under UV and visible light, and is considered the most promising multifunctional photocatalyst for CO_2_ conversion and water splitting [66,67].

ROS-mediated enzyme deactivation and sluggish electron transfer rate severely constrain the catalytic efficiency of artificial photosynthetic hybrids. In natural photosynthesis, the separation of reduction and photoexcitation protects the enzyme. By mimicking natural photosynthesis, Tian et al. designed a compartmentalized photocatalyst−enzyme hybrid for the conversion of CO_2_ to formic acid [68]. They conjugated the *2,2′-bipyridine-3,3′-dicarboxylic acid-Cl_2_ (Cp*Rh cocatalyst) complex with thiophene functionalized g-C_3_N_4_ (TPE-C_3_N_4_). The g-C_3_N_4_ is a promising photosensitizer that enables NADH regeneration under visible light and possesses extraordinary biocompatibility and chemical stability. To improve electron−hole recombination, TPE-g-C_3_N_4_ was used instead of the bulk g-C_3_N_4._ This hybrid exhibited 2.33 times higher NADH regeneration rate (9.33 μM/min) than the homogeneous hybrid. Tightly conjugated structure accelerated the electron transfer from TPE-C_3_N_4_ to the cocatalyst complex. To protect from photodamage, the formate dehydrogenase enzyme was encapsulated by coating with MOF. MAF-7, a zeolitic imidazolate complex prepared by coordinating Zn^2+^ ions and 3-methyl-1,2,4-triazole, was used as a MOF material. This compartmentalized the FDH from the destructive photoexcitation process. The MOF layer mimicked the function of the thylakoid membrane found in the chloroplast. The hydrophilic triazole linkers in MOF 7 stabilized the pH conditions and established a safe and stable microenvironment for FDH. The enhanced enzyme stability favored higher CO_2_ to formate conversion efficiency. After 9 h of exposure to light, this photo-biocatalyst system yielded 16.75 mM formic acid, which was estimated to be 3.24-fold greater than the homogeneous reaction system. Notably, the synergy between accelerated electron transfer from TPE-C_3_N_4_, regeneration of NADH, and protection of FDH by the compartmentalized system resulted in higher formic acid yield via CO_2_ conversion.

Non-toxic, low-cost, and efficient water splitting hybrid photocatalysts are useful for harnessing the full potential of AP. Tremblay et al. developed an NBH by integrating an ezyme-g-C_3_N_4_ photocatalyst with a non-photosynthetic bacterium and explored water splitting and PHB (bioplastic) production [69]. The system utilized a pure bovine liver catalase enzyme for H_2_O_2_ degradation and *R. eutropha* for bioplastic production. H_2_O_2_-degrading catalase can split water under sunlight and deliver reducing equivalents useful for microbial CO_2_ conversion. The g-C_3_N_4_-catalase photocatalyst exhibited a 3.4% solar-to-H_2_ conversion efficiency with the maximum H_2_ evolution rate of 55.72 mmol/h and stoichiometric O_2_ release. Meanwhile, the NBH photosynthesis system (g-C_3_N_4_-catalase- *R. eutropha*) increased the PHB production by 2 and 1.8 times from CO_2_ and fructose, respectively. Catalases enabled g-C3N4-mediated water splitting were used to drive the PHB production. The synergistic interface formed between enzymes, non-metallic photocatalysts, and bacteria served as the driving force for the conversion of CO_2_ into multi-carbon chemicals. Further optimization is required to comprehensively understand the mechanisms involved in this system.

**Figure 6:**
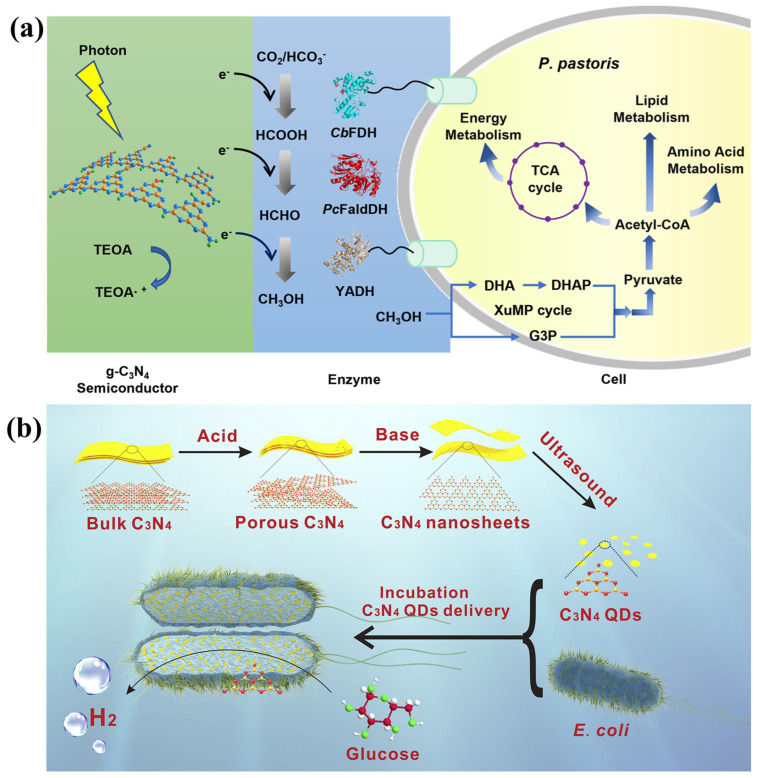
Carbon nanomaterial (g-C_3_N_4_)-based semiconductor-enzyme-cell hybrid system for energy conversion and CO_2_ reduction. (**a**) Enzyme cascade-g-C_3_N_4_ hybrid systems were used for the in situ transformation of methanol to biomass, surface displayed enzyme cascades with g-C_3_N_4_ to create a semiconductor–enzyme–cell hybrid system [70]. (**b**) Fabricating C3N4/E. coli biohybrid system for intracellular solar H_2_ production [71]. Figures reproduced with permission from cited references.

To achieve effective conversion of CO_2_ into multi-carbon organic compounds under visible light, Sheng et al. designed an enzyme-coupled hybrid catalyst (Figure 6a) [70]. The classical dehydrogenase enzymes were decorated on the biocompatible porous g-C_3_N_4_ nanosheets. Due to their high activity and expression, formate dehydrogenase, formaldehyde dehydrogenase, and alcohol dehydrogenase derived from *Candida boidinii* and *Pseudomonas cepacian* were coupled with g-C_3_N_4_. To enable mediator-free CO_2_ reduction in series, these enzymes were directly adsorbed on the surface of g-C_3_N_4_. Without an external electron mediator, this hybrid exhibited a significant quantum efficiency of 2.48% for the conversion of CO_2_ to methanol via enzyme-photocoupled reduction. The rate of CO_2_ reduction was 4.07 mg/(L·h), comparable to the typical photoelectrochemical cells and the photocatalyst coupled with electron mediators. Further, methanol was transported into the cell and used for biomass production by a semiconductor−enzyme−cell catalyst generated by depositing these enzymes on the porous g-C_3_N_4_ nanosheets-methanotrophic yeast, *Komagataella phaffiib* (*K. phaffiib)* hybrid. In particular, methanol imported into *K. phaffiib* cells was involved in the cellular metabolism, leading to the conversion of CO_2_ to biomass via the xylulose monophosphate cycle. This biocatalytic system can be used in a microbial cell for a sustainable, cost-effective conversion of CO_2_ to multi-carbon chemicals under solar radiation.

Wu et al. developed a non-metal intracellular NBH by anchoring g-C_3_N_4_ QDs (2–4 nm) on live *E. coli* cells and explored for solar light-induced H_2_ production (Figure 6b) [71]. The hybrid was prepared by inoculating *E. coli* with g-C_3_N_4_ QDs supplemented with growth medium. Greater biocompatibility and proper size g-C_3_N_4_ QDs made easy entry into *E. coli* cells. The light absorption ability of *E. coli* covered the UV to visible region, and NADH and Flavins were identified as responsible for photo-response. NADH in *E. coli* cells was attributed to the increase in H_2_ production under light. Due to greater separation and transfer of photogenerated electrons at the g-C_3_N_4_ QDs/NAD^+^ junction created at the inorganic-bio interface, the rate of H_2_ production reached 7800 ± 12 µmol/g·h, estimated as 77% higher than typical hybrids reported. In the dark, living *E. coli* cells produced 480 ± 8 µmol of H_2_ in 5 h, and it turned out to be 890 ± 10 µmol under simulated solar radiation. When studying for 50 h dark/light cycles, there were no obvious losses in the generation of H_2_. This work sheds light on the advantages of intracellular inorganic NBH systems for sunlight-to-fuel conversion.

The lack of utilization of photogenerated electrons results in lower yield and selectivity of methanogens and nanoscale semiconductor hybrids. Methanogens can survive in complex environments and possess unique self-repair and replication capabilities. However, there were mismatches between electron generation by the semiconductor and utilization in the metabolic process of the methanogen. To address these issues, Hu et al. integrated the cyanamide functionalized metal-free g-C_3_N_4_ with *M. barkeri* [72]. This hybrid bearing g-C_3_N_4_ and *M. barkeri* was self-assembled and combined via electrostatic interactions. When exposed to light, this self-assembled hybrid exhibited 93.4% selectivity for CH_4_ and a quantum yield of 50.3%, outperforming typical hybrids used for CO_2_ reduction. The distinct storage and redistribution of photogenerated electrons at the microbe-semiconductor interface allowed for the harnessing of excess electrons and their release when required. The robust conductance and capacitance of g-C_3_N_4_ prevented electron loss due to side reactions (H_2_ production) and electron–hole recombination. The cyanamide group facilitated electron storage and distribution at the biotic–abiotic interface and increased the number of molecular docking sites in g-C_3_N_4_. The surface area and CO_2_ adsorption ability of this hybrid were higher than that of the *M. barkeri*-CdS hybrid. The insights gained from this work can be used for designing metal-free semiconductor-based NBHs for CO_2_ to fuel conversion.

Protection of NBHs against the destructive UV portion of sunlight is very important for effective solar energy conversion. Gu et al. encapsulated microorganisms within the natural luminogens to shield them against high-energy UV photons [73]. Luminogens possess the aggregation-induced emission (AIE) property. They prepared *M. barkeri*-g-C_3_N_4_ hybrid and used natural berberine (BBR) as a protective layer. *M. barkeri* has a widespread presence in the environment and is known for its physiological and metabolic diversity. This hybrid was evaluated for the conversion of CO_2_ to methane performed under UVA irradiation. The g-C_3_N_4_ was synthesized by polymerizing melamine and decorated on the *M. barkeri*; then, BBR was assembled on the surface of the hybrid via self-precipitation. Under simulated solar light, the BBR encapsulated hybrid demonstrated 2.75 times higher methane production. Mechanistic insights revealed that BBR functioned as a UV sunscreen for the hybrid by converting the short-wavelength radiation into long-wavelength radiation. This process prevented the accumulation of ROS and protected g-C_3_N_4_ from photocorrosion. Additionally, BBR regulated the production and utilization of photoelectrons, enhancing the intracellular energy formation. These findings indicate that BBR, in addition to its UV-protective function, also serves as a photoenergist, contributing to the growth and metabolism of *M. barkeri.* The insights gained from this study can be applied to the large-scale conversion of CO_2_ into biofuels, which requires a high-energy light source.

Based on the above discussion, it is evident that g-C_3_N_4_ possesses strong potential for forming metal-free semiconductor-based nano-biohybrids (NBHs). Equal emphasis has been provided to construct both enzyme-based and microorganism-based NBHs, enabling effective solar-driven CO_2_-to-chemical conversion and H_2_ production. Improvements were noticed in the synthesis of formic acid [68], bioplastics [69], multi-carbon compounds [70], hydrogen [71,72], and methane [72,73]. The g-C_3_N_4_ QDs led to the formation of intracellular hybrids with *E. coli*.

The synergistic interface formed by g-C_3_N_4_ between enzymes and microbes provides an efficient driving force for charge transfer, enhancing photocatalytic performance. Particularly significant is the g-C_3_N_4_–M. barkeri hybrid [72], which demonstrated an exceptional quantum yield of 50.3% for CO_2_-to-methane conversion, substantially higher than typical hybrids. Incorporating UV-protective layers has also emerged as a promising strategy to maximize the capture and utilization of the entire solar spectrum, including its UV component. Nevertheless, further optimization and mechanistic studies are essential to fully understand the operational principles of g-C_3_N_4_-based NBHs.

## 6. Organic Polymer/Semiconductor–Microorganism Hybrids

Similar to natural photosynthetic pigments such as chlorophyll, carotenoids, and phycobilin, the conjugated and delocalized π-bond electrons contribute to the semiconducting properties of conjugated polymers and organic semiconductors. Unlike layered g-C_3_N_4_, conjugated polymers are linear compounds. Surface functionalization via side chains allows turning properties such as water solubility, biocompatibility, and binding with microorganisms. Conjugated polymers perform dual roles as light-harvesting antennas and interfacial electron bridges connecting microorganisms and materials. The optoelectronic properties of these polymers, integrated with the catalytic properties of microorganisms, function as AP systems for photosynthetic CO_2_ conversion, hydrogen production, and nitrogen fixation [74].

Gai et al. pioneered the development of a solar energy harvesting organic semiconductor-microorganism hybrid for CO_2_ conversion [75]. They coated *M. thermoacetica* with p-n heterojunction photosensitizers, using a derivative of perylene diimide and poly(fluorene-co-phenylene) to form the p-n heterojunction layer. This p-n coating enabled a high electron-hole separation. The p-type conjugated semiconductors are known for their high light-harvesting ability, bio-affinity, and biocompatibility. Due to electrostatic and hydrophobic interactions, the cationic side chains in the polymeric semiconductor intercalate well with the bacterial cell membrane, facilitating efficient electron transfer. This unique interaction between the light-harvesting p-n heterojunction layer and the bacteria is expected to circumvent the additional energy consumption required for transmembrane transport of redox shuttles. The transfer of photogenerated electrons from the p-n heterojunction layer to *M. thermoacetica* drives the Wood–Ljungdahl pathway, resulting in the conversion of CO_2_ to acetic acid. The efficiency of this hybrid (1.6%) is comparable to that of inorganic–microorganism hybrids.

**Figure 7:**
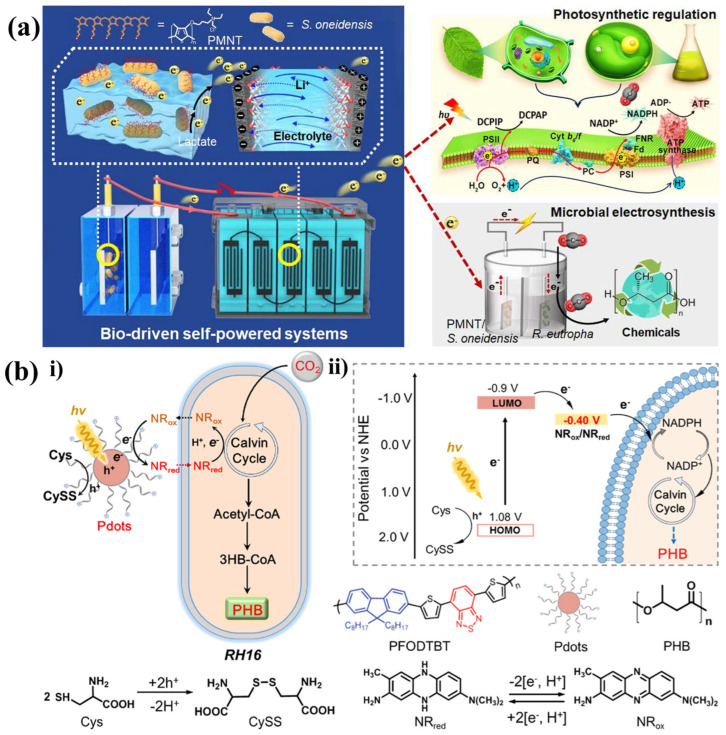
Complex augmentation of bio-driven self-powered systems integrating polymers with photosynthetic microbial systems. (**a**) Microbial electrosynthesis process developed by combining energy storage supercapacitors with energy conversion systems, hybrid S. oneidensis/poly[3-(3′-N,N,N-triethylamino-1′-propyloxy)-4-methyl-2,5-thiophene chloride] (PMNT) biofilms were utilized for microbial fuel cells assembly and CO_2_ fixation [76]. (**b**) Photosynthetic biohybrid factory comprising *R. eutropha H16*/NR/Pdots was used for CO_2_ reduction into PHB in *R. eutropha H16*. The PNM (Pdots) promotes the PHB production of *R. eutropha H16* biohybrids [77].

A strategy integrating electroactive microorganisms and electrochemical devices was developed to create a self-powered hybrid operating without an external energy supply. Microbial fuel cells generate electrical energy from biowaste sources. The electrogenic bacteria *S. oneidensis* can harvest electricity from organic biomass and transport it to anodes via extracellular electron transfer. This ensures sustainable operation, allowing self-powered devices to deliver electricity and light, eliminating the need for an external energy supply. Supercapacitors store and supply energy on demand. Chen et al. adopted this strategy to prepare a hybrid of polymeric semiconductor, poly [3-(3′-N,N,N-triethylamino-1′-propyloxy)-4-methyl-2,5-thiophene chloride] (PMNT) and *S. oneidensis* (Figure 7a) [76]. PMNT films are used in microbial fuel cells to generate electrocatalytic biocurrent from waste sources. This self-powered hybrid is capable of electrical energy generation, conversion, and storage. By facilitating energy storage in supercapacitors, energy derived from the metabolic processes of this electroactive microorganism can be used for long-term and continuous electricity production. The stored electrical energy is utilized for photosynthetic regulation and sustainable chemical production. A series of supercapacitors can be adapted to power LEDs, enabling photosynthetic regulation-mediated biomass conversion without exposure to external irradiation. Electricity-driven CO_2_ fixation can be performed for sustained chemical production when the self-powered system is switched off. This work integrates the production of renewable energy and chemicals, providing an on-demand, controllable energy production system with great potential for industrial applications.

The efficiency of CO_2_ conversion in the organic semiconductor–microorganism hybrid is limited by the lack of direct electron transport proteins in microorganisms. Yu et al. addressed this challenge by designing a hybrid system that facilitates transmembrane transport of semiconductor-generated electrons across the microbial cell wall (Figure 7a) [77]. In their approach, biocompatible polymer dots (Pdots, 70 nm) were combined with *Ralstonia eutropha H16* bacteria and Nile Red (NR), with Pdots and NR serving as photosensitizers and electron-shuttling agents, respectively. Water-soluble Pdots were synthesized via nanoprecipitation of a hydrophobic conjugated polymer, poly(N,N-dimethylaminoethyl methacrylate)-poly(9,9-dihexy-2,7-fluorene)-poly(N,N-dimethylaminoethyl methacrylate), followed by grafting with carboxyl-functionalized ethylene oxide-grafted polystyrene. This hybrid system was utilized for the microbial production of poly-3-hydroxybutyrate (PHB), a biodegradable and mechanically robust intracellular polyester. Upon light exposure, the Pdots adhered to the surface of *R. eutropha H16*, generating electrons and holes. The holes were consumed by Cys, while the electrons reduced NR and diffused abundantly across the microbial cell membrane. Within *R. eutropha H16*, these electrons enhanced nicotinamide adenine dinucleotide phosphate (NADPH) production and activated the Calvin cycle, enabling CO_2_ fixation into PHB. By leveraging NR, photogenerated electrons amplified NADPH production and further facilitated the Calvin cycle, driving CO_2_ conversion to PHB. This system achieved a yield of 21.3 ± 3.78 mg/L, approximately three-fold higher than that of *R. eutropha H16* alone. The successful implementation of this ternary synergistic biochemical factory demonstrates its potential for solar-driven, renewable production of valuable chemicals.

The above discussion highlights that tunable surface interactions and lower toxicity enable the effective integration of polymeric nanomaterials with microorganisms through non-covalent attachment and membrane-bound interactions, largely resulting in extracellular NBHs. These systems have been successfully employed for CO_2_ conversion and H_2_ production. Notably, some of these NBHs operate as self-powered biochemical factories, driving solar-powered renewable chemical synthesis. However, for practical and scalable applications, there are associated challenges such as the long-term stability of polymeric materials, structural complexity, economic viability, and large-scale integration.

Utschig et al. investigated intermolecular electron transfer in a H_2_-producing chlorophyll system involving a natural photosystem I (PSI)–platinum nanoparticles (Pt NPs) hybrid (PSI-Pt) [78]. PSI and Pt were linked via electrostatic interactions, with Cytochrome C6 serving as a mediator and ascorbate acting as a sacrificial electron donor. Mercaptosuccinic acid was employed to stabilize Pt NPs (∼3.0 nm in size). The hybrid was created by mixing 1.2–2 mol equivalents of Pt NPs with native PSI derived from *Synechococcus leopoliensis* or *Synechococcus lividus*. A liquid medium consisting of a buffer (pH 8.0) prepared using 20 mM Tris-Cl and 0.04% Triton was used during the process. Unbound Pt NPs were removed to yield the PSI-Pt hybrid, which commenced H_2_ production upon exposure to visible light emitted by a Xenon lamp. The PSI-Pt hybrid achieved a remarkable H_2_ production rate of 244 μmol H_2_/(mg chlorophyll)·h, with corresponding turnover numbers exceeding 20,000 (mol H_2_/mol PSI)/h—substantially higher than rates observed in sensitizer–cobaloxime conjugate-based photochemical systems. These results demonstrated that self-assembled, non-covalent photocatalyst complexes can effectively enable visible light-driven H_2_ production without requiring molecular wiring to connect PSI, the [4Fe-4S] cluster FB, and Pt NPs.

Holá et al. explored the self-assembly of highly fluorescent aspartic acid and carbon dots (CDs) as photosensitizers for light-driven H_2_ production facilitated by the [FeFe] hydrogenase enzyme derived from *Chlamydomonas reinhardtii* [79]. They systematically studied the photosensitizer assembly to understand the dynamics and influence of electron transfer on photocatalytic efficiency. Sacrificial electron donors generated favorable electrostatic conditions that significantly enhanced the overall photocatalytic performance. Under these optimal electrostatic conditions, light-driven (420 nm) H_2_ production achieved a quantum efficiency of 1.7%. The hybrid assembly also demonstrated stability, maintaining its performance for more than a week.

Yang et al. utilized conjugated polymer NPs as photosensitizers to create hybrids with *Escherichia coli* (*E. coli*) [80]. This photobiocatalytic NBH was constructed by electrostatically assembling linear conjugated polymer NPs onto *E. coli* cells. The *E. coli* was genetically engineered to express [FeFe]-hydrogenase along with endogenous [NiFe]-hydrogenase. Conjugated polymers were employed to modulate surface properties, enhancing the proton reduction efficiency of the biohybrids. This functional synergy resulted in efficient H_2_ production under simulated solar light. A representative hybrid, consisting of a conjugate copolymer of fluorene and dibenzo[b,d]thiophene sulfone assembled with *E. coli*, exhibited substantial H_2_ production under simulated solar light. The system achieved an H_2_ production rate of 3.442 mmol/g·h, which was approximately 30 times higher than the rate achieved by the conjugated polymer photocatalyst alone (0.105 mmol/g·h). Notably, H_2_ production was undetectable when *E. coli* was used independently, underscoring the critical synergy between the polymer NPs and the microbial cells. The authors emphasized the advantages of organic semiconductors over inorganic materials, including their solution processability, low toxicity, and tunable surface interactions with microbial cells. These features highlight the potential of organic semiconductors in developing efficient biohybrid systems for renewable energy production.

## 7. Summary and Future Prospects

Advancements in NBH-based artificial photosynthetic (AP) systems focus on creating viable pathways for storing solar energy within chemical bonds, emphasizing the need for high chemical conversion and quantum efficiency. Various NBHs have been engineered to function as light-absorbing heterotrophic microbes by integrating conventional inorganic and organic nanomaterials (NMs) with bacteria, yeast, and enzymes. Mostly, microbial cells of *M. thermoacetica*, *M. barkeri*, *S. ovata*, *S. cerevisiae, R. eutropha*, and genetically engineered *E. coli* were used in constructing NBHs. Conversely, CdS, CdSe QDs, Au NCs, SiO_2_ NWs, and TiO_2_ NPs were widely used as PNMs. Organic photosensitizers such as g-C_3_N_4_, perylene diimide and poly(fluorene-co-phenylene), poly[3-(3′-N,N,N-triethylamino-1′-propyloxy)-4-methyl-2,5-thiophene chloride], poly(N,N-dimethylamino ethyl methacrylate)-poly(9,9-N-dihyxyl-2,7-fluorene)-poly(N,N-dimethylamino ethyl methacrylate, and 2,2′-bipyridine-3,3′-dicarboxylic acid-Cl_2_ were also used for preparing NBHs. Various material forms, including NPs, QDs, NCs, NWs, and Pdots, were employed. Together with CdS, AglnS_2_/In_2_S_3_, and CuInS_2_/ZnS were also used for H_2_ generation. In some studies, biological photosensitizers such as bacteriorhodopsin were combined with PNMs. There were also AP systems that directly combined enzymes with photocatalysts. Several dehydrogenase enzymes, especially formate and formaldehyde dehydrogenase, have received significant attention. NBH-based AP systems successfully delivered a range of simple and value-added chemicals, including PHB, acetate, formate, methane, methanol, ethanol, n-butanol, and glycerol.

A large number of extracellular NBHs were reported compared to intracellular NBHs. Among the intracellular NBHs, the periplasmic NBHs were given importance, due to their high photoconversion rate compared to extracellular NBHs. Visible light-harvesting NBHs were predominantly observed. The destructive effect of cytotoxicity originating from nanomaterials (NMs) was addressed by in vivo generation of NMs using cellular elemental sources, biocompatible coatings, and organic semiconductors. Use of Cys as a sulfur source for CdS is a typical example of in vivo synthesis of nanomaterials.

While nanotechnology has enabled the creation of efficient, scalable, and robust NBH-based AP systems, the commercialization of AP-derived fuels remains an unmet challenge. Fundamental challenges remain as bottlenecks in transitioning from laboratory-scale research to commercial applications. Owing to their interdisciplinary nature, NBHs require both theoretical and technical backgrounds encompassing biology, material science, and electrochemistry. The process of knowledge and technology amalgamation delays the realization of sufficient fundamental challenges. It is well understood that in NBHs, PNMs undergo surface photoexcitation when exposed to light. This photoexcitation generates electron–hole pairs. Then the electrons enter microbial cells, and generate energy carriers (NADPH and ATP), promoting the synthesis of value-added chemicals from CO_2_ and H_2_ from water.

In most reports, greater importance is given to electron transfer, while the fate of holes is not discussed with equal importance. Effective transfer of photogenerated electrons from PNMs to microbial cells or enzymes determines the efficiency of the AP system. The timely removal of photogenerated holes is crucial for the successful operation of the AP system. Otherwise, holes recombine with electrons and cause photocorrosion-induced damage to PNMs.

Moreover, since microbes are the workhorses of NBH-based AP systems, they must be kept alive for sustainable use. In photocatalysis, holes are used for generating ROS to eliminate microbes through the oxidative damage of their cell membrane. Conversely, in NBHs, the photogenerated holes must be scavenged immediately to protect the microbes. If the photogenerated holes are not handled properly, the performance of NBHs can significantly decrease or become non-functional. As basic mechanistic insights are required to fully understand NBH-based AP systems, the fate of photogenerated holes must be well accounted for in future investigations.

The following aspects can be considered for future research:

1. Synergistic Microbial Consortia: Microorganisms thrive as diverse communities. In a shared living environment, different species of microbes exchange metabolic byproducts. Therefore, a synergistic consortium of microbial cells can be combined with PNMs to achieve an effective natural electron transfer pathway and high quantum efficiency.
2. Stacking Different PNMs: Stacking different PNMs capable of absorbing various components of solar radiation can enhance light harvesting. Thus, efforts to create NBHs with stacked PNMs need to be increased.
3. Nitrogen Fixation: While NBHs have been used for light-driven conversion of CO_2_ into chemicals and the light-driven splitting of water into H_2_ and O_2_, limited attention has been given to nitrogen fixation. This process is critical for the production of carbon-neutral fuels.
4. Impact of Positively Charged PNMs: The impact of negatively charged PNMs has been extensively explored, but the effect of positively charged PNMs on the cellular environment remains largely unexplored. Future studies should focus on this aspect.
5. Organic Photosensitizers: Recent works have given limited attention to the use of dyes and polymeric photosensitizers. These organic molecules can diffuse inside microbial cells, facilitating the formation of efficient intracellular hybrids.
6. Climatic Adaptability: Although NBH-based AP has been successfully explored for chemical and fuel production, the full potential of sunlight-to-chemical conversion has not yet been achieved, with benchmark efficiency. NBHs that function under various climatic conditions must be developed.
7. Bio-inspired NBHs: Developing a variety of bio-inspired NBHs that mimic natural photosynthetic structures (e.g., chloroplasts) requires greater insights into light and electron utilization.
8. Dynamic Interfacing: Developing stimuli-responsive semiconductors that adjust electron output based on microbial metabolic demand is essential. Integrating biosensors with dynamic interfaces could enable autonomous adjustments. For example, lactate sensors in NBHs could trigger a pH-responsive polymer to release electrons only when product concentrations are low.

Current research efforts are directed toward refining the interface between nanomaterials and biological components to improve the stability, efficiency, and scalability of NBHs. The exploration of novel NMs and bioinspired designs presents exciting opportunities for advancing next-generation AP technology with enhanced capabilities for sustainable fuel production. In summary, the integration of nanotechnology and biological systems in NBHs holds great potential for AP. Ongoing studies continue to address existing challenges, aiming to optimize performance and accelerate the transition toward practical applications.

## Data Availability

The original contributions presented in this study are included in the article. Further inquiries can be directed to the corresponding authors.

## References

[1] Blankenship R.E., Tiede D.M., Barber J., Brudvig G.W., Fleming G., Ghirardi M., Gunner M.R., Junge W., Kramer D.M., Melis A. (2011). Comparing Photosynthetic and Photovoltaic Efficiencies and Recognizing the Potential for Improvement. Science.

[2] Ji W., Liu J., Sha C., Yong Y.-C., Jiang Y., Fang Z. (2024). Nanomaterial-Biological Hybrid Systems: Advancements in Solar-Driven CO2-to-Chemical Conversion. Green Carbon.

[3] Okoro G., Husain S., Saukani M., Mutalik C., Yougbaré S., Hsiao Y.C., Kuo T.R. (2023). Emerging Trends in Nanomaterials for Photosynthetic Biohybrid Systems. ACS Mater. Lett..

[4] Liang J., Xiao K., Wang X., Hou T., Zeng C., Gao X., Wang B., Zhong C. (2024). Revisiting Solar Energy Flow in Nanomaterial-Microorganism Hybrid Systems. Chem. Rev..

[5] Barber J. (2009). Photosynthetic Energy Conversion: Natural and Artificial. Chem. Soc. Rev..

[6] Lewis N.S. (2016). Developing a Scalable Artificial Photosynthesis Technology through Nanomaterials by Design. Nat. Nanotechnol..

[7] Fujishima A., Honda K. (1972). Electrochemical Photolysis of Water at a Semiconductor Electrode. Nature.

[8] Sanchez R.J., Srinivasan C., Munroe W.H., Wallace M.A., Martins J., Kao T.Y., Le K., Gralla E.B., Valentine J.S. (2005). Exogenous Manganous Ion at Millimolar Levels Rescues All Known Dioxygen-Sensitive Phenotypes of Yeast Lacking CuZnSOD. J. Biol. Inorg. Chem..

[9] Cazelles R., Drone J., Fajula F., Ersen O., Moldovan S., Galarneau A. (2013). Reduction of CO_2_ to Methanol by a Polyenzymatic System Encapsulated in Phospholipids-Silica Nanocapsules. New J. Chem..

[10] Kumar M., Sahoo P.C., Parida K. (2024). Minireview on Microbe-Nanomaterial Synergies: Fundamental, Interface Mechanism, Characterization and CO_2_ Photo-Fixation. Results Surf. Interfaces.

[11] Li L., Xu Z., Huang X. (2021). Whole-Cell-Based Photosynthetic Biohybrid Systems for Energy and Environmental Applications. Chempluschem.

[12] Shen J., Liu Y., Qiao L. (2023). Photodriven Chemical Synthesis by Whole-Cell-Based Biohybrid Systems: From System Construction to Mechanism Study. ACS Appl. Mater. Interfaces.

[13] Sakimoto K.K., Kornienko N., Yang P. (2017). Cyborgian Material Design for Solar Fuel Production: The Emerging Photosynthetic Biohybrid Systems. Acc. Chem. Res..

[14] Sakimoto K.K., Kornienko N., Cestellos-Blanco S., Lim J., Liu C., Yang P. (2018). Physical Biology of the Materials-Microorganism Interface. J. Am. Chem. Soc..

[15] Cestellos-Blanco S., Zhang H., Kim J.M., Shen Y., Yang P. (2020). Photosynthetic Semiconductor Biohybrids for Solar-Driven Biocatalysis. Nat. Catal..

[16] Dogutan D.K., Nocera D.G. (2019). Artificial Photosynthesis at Efficiencies Greatly Exceeding That of Natural Photosynthesis. Acc. Chem. Res..

[17] Yang P. (2021). Liquid Sunlight: The Evolution of Photosynthetic Biohybrids. Nano Lett..

[18] Zhou X., Zeng Y., Lv F., Bai H., Wang S. (2022). Organic Semiconductor-Organism Interfaces for Augmenting Natural and Artificial Photosynthesis. Acc. Chem. Res..

[19] Yu Y., Guo S., Lv S., Tian R., Cheng S., Chen Y. (2024). Eradicating the Photogenerated Holes in a PhotoAP systems-Microbe Hybrid System: A Review. ACS Appl. Mater. Interfaces.

[20] Xiao S., Li Z., Fu Q., Li Y., Li J., Zhang L., Liao Q., Zhu X. (2020). Hybrid Microbial Photoelectrochemical System Reduces CO_2_ to CH4 with 1.28% Solar Energy Conversion Efficiency. Chem. Eng. J..

[21] Lu X. (2010). A Perspective: Photosynthetic Production of Fatty Acid-Based Biofuels in Genetically Engineered Cyanobacteria. Biotechnol. Adv..

[22] Srirangan K., Pyne M.E., Perry Chou C. (2011). Biochemical and Genetic Engineering Strategies to Enhance Hydrogen Production in Photosynthetic Algae and Cyanobacteria. Bioresour. Technol..

[23] Lin Y., Shi J., Feng W., Yue J., Luo Y., Chen S., Yang B., Jiang Y., Hu H., Zhou C. (2023). Periplasmic Biomineralization for Semi-Artificial Photosynthesis. Sci. Adv..

[24] Woolerton T.W., Sheard S., Reisner E., Pierce E., Ragsdale S.W., Armstrong F.A. (2010). Efficient and Clean Photoreduction of CO_2_ to CO by Enzyme-Modified TiO_2_ Nanoparticles Using Visible Light. J. Am. Chem. Soc..

[25] Brown K.A., Wilker M.B., Boehm M., Hamby H., Dukovic G., King P.W. (2016). Photocatalytic Regeneration of Nicotinamide Cofactors by Quantum Dot-Enzyme Biohybrid Complexes. ACS Catal..

[26] Sweeny R.Y., Mao C., Gao X., Burt J.L., Belcher A.M., Georgiou G., Iverson B.L. (2004). Bacterial biosynthesis of cadmium sulfide nanocrystals. Chem. Biol..

[27] Kowshik M., Deshmukh N., Vogel W., Urban J., Kulkarni S.K., Paknikar K.M. (2002). Microbial Synthesis of Semiconductor CdS Nanoparticles, Their Characterization, and Their Use in the Fabrication of an Ideal Diode. Biotechnol. Bioeng..

[28] Wang J., Chen N., Bian G., Mu X., Du N., Wang W., Ma C.G., Fu S., Huang B., Liu T. (2022). Solar-Driven Overproduction of Biofuels in Microorganisms. Angew. Chem.-Int. Ed..

[29] Cheng L., Xiang Q., Liao Y., Zhang H. (2018). CdS-Based Photocatalysts. Energy Environ. Sci..

[30] Sakimoto K.K., Wong A.B., Yang P. (2016). Self-Photosensitization of Nonphotosynthetic Bacteria for Solar-to-Chemical Production. Science.

[31] Kornienko N., Sakimoto K.K., Herlihy D.M., Nguyen S.C., Alivisatos A.P., Harris C.B., Schwartzberg A., Yang P. (2016). Spectroscopic Elucidation of Energy Transfer in Hybrid Inorganic-Biological Organisms for Solar-to-Chemical Production. Proc. Natl. Acad. Sci. USA.

[32] Ding Y., Bertram J.R., Eckert C., Bommareddy R.R., Patel R., Conradie A., Bryan S., Nagpal P. (2019). Nanorg Microbial Factories: Light-Driven Renewable Biochemical Synthesis Using Quantum Dot-Bacteria Nanobiohybrids. J. Am. Chem. Soc..

[33] Ye J., Yu J., Zhang Y., Chen M., Liu X., Zhou S., He Z. (2019). Light-Driven Carbon Dioxide Reduction to Methane by Methanosarcina Barkeri-CdS Biohybrid. Appl. Catal. B.

[34] Kumar M., Sahoo P.C., Srikanth S., Bagai R., Puri S.K., Ramakumar S.S.V. (2019). Photosensitization of Electro-Active Microbes for Solar Assisted Carbon Dioxide Transformation. Bioresour. Technol..

[35] Zhang S., Shi J., Sun Y., Wu Y., Zhang Y., Cai Z., Chen Y., You C., Han P., Jiang Z. (2019). Artificial Thylakoid for the Coordinated Photoenzymatic Reduction of Carbon Dioxide. ACS Catal..

[36] Wang B., Jiang Z., Yu J.C., Wang J., Wong P.K. (2019). Enhanced CO_2_ Reduction and Valuable C2+ Chemical Production by a CdS-Photosynthetic Hybrid System. Nanoscale.

[37] Xu M., Tremblay P.L., Ding R., Xiao J., Wang J., Kang Y., Zhang T. (2021). Photo-Augmented PHB Production from CO_2_ or Fructose by Cupriavidus Necator and Shape-Optimized CdS Nanorods. Sci. Total. Environ..

[38] He Y., Wang S., Han X., Shen J., Lu Y., Zhao J., Shen C., Qiao L. (2022). Photosynthesis of Acetate by Sporomusa Ovata-CdS Biohybrid System. ACS Appl. Mater. Interfaces.

[39] Wang B., Zeng C., Chu K.H., Wu D., Yip H.Y., Ye L., Wong P.K. (2017). Enhanced Biological Hydrogen Production from Escherichia Coli with Surface Precipitated Cadmium Sulfide Nanoparticles. Adv. Energy Mater..

[40] Chen M.Y., Fang Z., Xu L.X., Zhou D., Yang X.J., Zhu H.J., Yong Y.C. (2021). Enhancement of Photo-Driven Biomethanation under Visible Light by Nano-Engineering of Rhodopseudomonas Palustris. Bioresour. Bioprocess..

[41] Liang G., Xu X., Chen X., Wu J., Song W., Wei W., Liu J., Li X., Liu L., Gao C. (2023). Designing a Periplasmic Photosynthetic Biohybrid System for Succinate and Electric Energy Production. Chem. Eng. J..

[42] Wei W., Sun P., Li Z., Song K., Su W., Wang B., Liu Y., Zhao J. (2018). A Surface-Display Biohybrid Approach to Light-Driven Hydrogen Production in Air. Sci. Adv..

[43] Gao Y., Wu J., Xia Q., Liu J., Zhu J.J., Zhang J.R., Chen X., Zhu W., Chen Z. (2024). Operando Spectroscopic Elucidation of the Bubble Sunshade Effect in Inorganic-Biological Hybrids for Photosynthetic Hydrogen Production. ACS Nano.

[44] Yang C.C., Yu Y.H., Van Der Linden B., Wu J.C.S., Mul G. (2010). Artificial Photosynthesis over Crystalline TiO_2_-Based Catalysts: Fact or Fiction?. J. Am. Chem. Soc..

[45] Yuan L., Han C., Pagliaro M., Xu Y.J. (2016). Origin of Enhancing the Photocatalytic Performance of TiO_2_ for Artificial Photoreduction of CO_2_ through a SiO_2_ Coating Strategy. J. Phys. Chem. C.

[46] Ruan X., Li S., Huang C., Zheng W., Cui X., Ravi S.K. (2024). Catalyzing Artificial Photosynthesis with TiO_2_ Heterostructures and Hybrids: Emerging Trends in a Classical yet Contemporary Photocatalyst. Adv. Mater..

[47] Liu C., Gallagher J.J., Sakimoto K.K., Nichols E.M., Chang C.J., Chang M.C.Y., Yang P. (2015). Nanowire-Bacteria Hybrids for Unassisted Solar Carbon Dioxide Fixation to Value-Added Chemicals. Nano Lett..

[48] Su Y., Cestellos-Blanco S., Kim J.M., Shen Y., Kong Q., Lu D., Liu C., Zhang H., Cao Y., Yang P. (2020). Close-Packed Nanowire-Bacteria Hybrids for Efficient Solar-Driven CO_2_ Fixation. Joule.

[49] Honda Y., Watanabe M., Hagiwara H., Ida S., Ishihara T. (2017). Inorganic/Whole-Cell Biohybrid Photocatalyst for Highly Efficient Hydrogen Production from Water. Appl. Catal. B.

[50] Chen Z., Zhang H., Guo P., Zhang J., Tira G., Kim Y.J., Wu Y.A., Liu Y., Wen J., Rajh T. (2019). Semi-Artificial Photosynthetic CO_2_ Reduction through Purple Membrane Re-Engineering with Semiconductor. J. Am. Chem. Soc..

[51] Kim J., Cestellos-Blanco S., Shen Y.X., Cai R., Yang P. (2022). Enhancing Biohybrid CO_2_ to Multicarbon Reduction via Adapted Whole-Cell Catalysts. Nano Lett..

[52] Zhao S., Liu Q., Li Y., Feng L., Fu S., Mazarji M., Pan J. (2024). The Role of Semi-Artificial Photosynthetic Systems in Energy and Environmental Solutions: A Critical Review. Biofuel Res. J..

[53] Surana K., Singh P.K., Rhee H.W., Bhattacharya B. (2014). Synthesis, Characterization and Application of CdSe Quantum Dots. J. Ind. Eng. Chem..

[54] Li X.B., Tung C.H., Wu L.Z. (2018). Semiconducting Quantum Dots for Artificial Photosynthesis. Nat. Rev. Chem..

[55] Tsuneda T., Ten-No S.L. (2022). Water-Oxidation Mechanism of Cobalt Phosphate Co-Catalyst in Artificial Photosynthesis: A Theoretical Study. Phys. Chem. Chem. Phys..

[56] Gautam A., Gore P.M., Kandasubramanian B. (2020). Nanocluster Materials in Photosynthetic Machines. Chem. Eng. J..

[57] Rahman A., Parwaiz S., Sohn Y., Mansoob Khan M. (2024). Advances in Artificial Photosynthesis: The Role of Chalcogenides and Chalcogenide-Based Heterostructures. ChemPhotoChem.

[58] Guo J., Suástegui M., Sakimoto K.K., Moody V.M., Xiao G., Nocera D.G., Joshi N.S. (2018). Light-Driven Fine Chemical Production in Yeast Biohybrids. Science.

[59] Chakraborty I.N., Jain V., Roy P., Kumar P., Vinod C.P., Pillai P.P. (2024). Photocatalytic Regeneration of Reactive Cofactors with InP Quantum Dots for the Continuous Chemical Synthesis. ACS Catal..

[60] Liu C., Colón B.C., Ziesack M., Silver P.A., Nocera D.G. (2016). Water Splitting–Biosynthetic System with CO_2_ Reduction Efficiencies Exceeding Photosynthesis. Science.

[61] Zhang H., Liu H., Tian Z., Lu D., Yu Y., Cestellos-Blanco S., Sakimoto K.K., Yang P. (2018). Bacteria Photosensitized by Intracellular Gold Nanoclusters for Solar Fuel Production. Nat. Nanotechnol..

[62] Jiang Z., Wang B., Yu J.C., Wang J., An T., Zhao H., Li H., Yuan S., Wong P.K. (2018). AglnS_2_/In_2_S_3_ Heterostructure Sensitization of Escherichia Coli for Sustainable Hydrogen Production. Nano Energy.

[63] Luo B., Wang Y.Z., Li D., Shen H., Xu L.X., Fang Z., Xia Z., Ren J., Shi W., Yong Y.C. (2021). A Periplasmic Photosensitized Biohybrid System for Solar Hydrogen Production. Adv. Energy Mater..

[64] Edwards E.H., Jelušić J., Kosko R.M., McClelland K.P., Ngarnim S.S., Chiang W., Lampa-Pastirk S., Krauss T., Bren K.L. (2023). Shewanella Oneidensis MR-1 Respires CdSe Quantum Dots for Photocatalytic Hydrogen Evolution. Proc. Natl. Acad. Sci. USA.

[65] Wang Y., Zhao Y., Wang S., Xiao G., Jin Y., Wang Z., Su H. (2023). Visible-Light-Driven Enhanced Biohydrogen Production by Photo-Biohybrid System Based on Photoelectron Transfer between Intracellular Photosensitizer Gold Nanoparticles and Clostridium Butyricum. ACS Sustain. Chem. Eng..

[66] Lu L., Lv Z., Si Y., Liu M., Zhang S. (2018). Recent Progress on Band and Surface Engineering of Graphitic Carbon Nitride for Artificial Photosynthesis. Appl. Surf. Sci..

[67] Cruz D., Żółtowska S., Savateev O., Antonietti M., Giusto P. (2025). Carbon Nitride Caught in the Act of Artificial Photosynthesis. Nat. Commun..

[68] Tian Y., Zhou Y., Zong Y., Li J., Yang N., Zhang M., Guo Z., Song H. (2020). Construction of Functionally Compartmental Inorganic Photocatalyst-Enzyme System via Imitating Chloroplast for Efficient Photoreduction of CO_2_ to Formic Acid. ACS Appl. Mater. Interfaces.

[69] Tremblay P.L., Xu M., Chen Y., Zhang T. (2020). Nonmetallic Abiotic-Biological Hybrid Photocatalyst for Visible Water Splitting and Carbon Dioxide Reduction. iScience.

[70] Sheng Y., Guo F., Guo B., Wang N., Sun Y., Liu H., Feng X., Han Q., Yu Y., Li C. (2023). Light-Driven CO_2_ Reduction with a Surface-Displayed Enzyme Cascade-C_3_N_4_ Hybrid. ACS Synth. Biol..

[71] Wu D., Zhang W., Fu B., Zhang Z. (2022). Living Intracellular Inorganic-Microorganism Biohybrid System for Efficient Solar Hydrogen Generation. Joule.

[72] Hu A., Ye J., Ren G., Qi Y., Chen Y., Zhou S. (2022). Metal-Free Semiconductor-Based Bio-Nano Hybrids for Sustainable CO_2_-to-CH_4_ Conversion with High Quantum Yield. Angew. Chem.-Int. Ed..

[73] Gu W., Hu J., Li L., Hong M., Yang C., Ren G., Ye J., Zhou S. (2024). Natural AIEgens as Ultraviolet Sunscreens and Photosynergists for Solar Fuel Production. Environ. Sci. Technol..

[74] Chen W., Lin H., Yu W., Huang Y., Lv F., Bai H., Wang S. (2024). Organic Semiconducting Polymers for Augmenting Biosynthesis and Bioconversion. JACS Au.

[75] Gai P., Yu W., Zhao H., Qi R., Li F., Liu L., Lv F., Wang S. (2020). Solar-Powered Organic Semiconductor–Bacteria Biohybrids for CO2 Reduction into Acetic Acid. Angew. Chem.-Int. Ed..

[76] Chen W., Yu W., Wang Z., Gao Z., Zhang M., Zhu C., Lv F., Huang Y., Bai H., Wang S. (2023). Self-Powered Biohybrid Systems Based on Organic Materials for Sustainable Biosynthesis. ACS Appl. Mater. Interfaces.

[77] Yu W., Pavliuk M.V., Liu A., Zeng Y., Xia S., Huang Y., Bai H., Lv F., Tian H., Wang S. (2023). Photosynthetic Polymer Dots-Bacteria Biohybrid System Based on Transmembrane Electron Transport for Fixing CO_2_ into Poly-3-Hydroxybutyrate. ACS Appl. Mater. Interfaces.

[78] Utschig L.M., Dimitrijevic N.M., Poluektov O.G., Chemerisov S.D., Mulfort K.L., Tiede D.M. (2011). Photocatalytic Hydrogen Production from Noncovalent Biohybrid Photosystem I/Pt Nanoparticle Complexes. J. Phys. Chem. Lett..

[79] Holá K., Pavliuk M.V., Németh B., Huang P., Zdražil L., Land H., Berggren G., Tian H. (2020). Carbon Dots and [FeFe] Hydrogenase Biohybrid Assemblies for Efficient Light-Driven Hydrogen Evolution. ACS Catal..

[80] Yang Y., Zwijnenburg M.A., Gardner A.M., Adamczyk S., Yang J., Sun Y., Jiang Q., Cowan A.J., Sprick R.S., Liu L.N. (2024). Conjugated Polymer/Recombinant Escherichia Coli Biohybrid Systems for Photobiocatalytic Hydrogen Production. ACS Nano.

